# A metabolomic approach to target antimalarial metabolites in the *Artemisia annua* fungal endophytes

**DOI:** 10.1038/s41598-021-82201-8

**Published:** 2021-02-02

**Authors:** Hani A. Alhadrami, Ahmed M. Sayed, Ahmed O. El-Gendy, Yara I. Shamikh, Yasser Gaber, Walid Bakeer, Noheir H. Sheirf, Eman Z. Attia, Gehan M. Shaban, Basma A. Khalifa, Che J. Ngwa, Gabriele Pradel, Mostafa E. Rateb, Hossam M. Hassan, Dalal H. M. Alkhalifah, Usama Ramadan Abdelmohsen, Wael N. Hozzein

**Affiliations:** 1 Department of Medical Laboratory Technology, Faculty of Applied Medical Sciences, King Abdulaziz University, Jeddah, 21589 Saudi Arabia; 2 Molecular Diagnostic Lab, King Abdulaziz University Hospital, King Abdulaziz University, P. O. Box 80402, Jeddah, 21589 Saudi Arabia; 3 Department of Pharmacognosy, Faculty of Pharmacy, Nahda University, Beni-Suef, 62513 Egypt; 4 Department of Microbiology and Immunology, Faculty of Pharmacy, Beni-Suef University, Beni-Suef, 62514 Egypt; 5 Department of Microbiology and Immunology, Nahda University, Beni-Suef, 62513 Egypt; 6 Department of Virology, Egypt Center for Research and Regenerative Medicine (ECRRM), Cairo, 11517 Egypt; 7 Department of Pharmaceutics and Pharmaceutical Technology, College of Pharmacy, Mutah University, Karak, 61710 Jordan; 8 Drug Radiation Research Department, National Center for Radiation Research and Technology, Atomic Energy Authority, Cairo, Egypt; 9 Department of Pharmacognosy, Faculty of Pharmacy, Minia University, Minia, 61519 Egypt; 10 Department of Botany and Microbiology, Faculty of Science, Minia University, Minia, 61519 Egypt; 11 Division of Cellular and Applied Infection Biology, Institute of Zoology, RWTH Aachen University, 52074 Aachen, Germany; 12 School of Computing, Engineering and Physical Sciences, University of the West of Scotland, Paisley, PA1 2BE UK; 13 Department of Pharmacognosy, Faculty of Pharmacy, Beni-Suef University, Beni-Suef, 62514 Egypt; 14 Biology Department, College of Science, Princess Nourah Bint Abdulrahman University, Riyadh, Saudi Arabia; 15 Department of Pharmacognosy, Faculty of Pharmacy, Deraya University, New 61111, Minia, Egypt; 16 Bioproducts Research Chair, Zoology Department, College of Science, King Saud University, Riyadh, Saudi Arabia; 17 Botany and Microbiology Department, Faculty of Science, Beni-Suef University, Beni-Suef, Egypt

**Keywords:** Drug discovery, Microbiology

## Abstract

Fungal endophytes are a major source of anti-infective agents and other medically relevant compounds. However, their classical blinded-chemical investigation is a challenging process due to their highly complex chemical makeup. Thus, utilizing cheminformatics tools such as metabolomics and computer-aided modelling is of great help deal with such complexity and select the most probable bioactive candidates. In the present study, we have explored the fungal endophytes associated with the well-known antimalarial medicinal plant *Artemisia annua* for their production of further antimalarial agents. Based on the preliminary antimalarial screening of these endophytes and using LC-HRMS-based metabolomics and multivariate analyses, we suggested different potentially active metabolites (compounds **1–8**). Further in silico investigation using the neural-network-based prediction software PASS led to the selection of a group of quinone derivatives (compounds **1–5)** as the most possible active hits. Subsequent in vitro validation revealed emodin (**1**) and physcion (**2**) to be potent antimalarial candidates with IC_50_ values of 0.9 and 1.9 µM, respectively. Our approach in the present investigation therefore can be applied as a preliminary evaluation step in the natural products drug discovery, which in turn can facilitate the isolation of selected metabolites notably the biologically active ones.

## Introduction

Pharmacologically active lead compound discovery campaigns are still depending on natural products, even when compared with modern tools and techniques such as high-throughput screening (HTS) of compounds obtained through chemical synthesis or combinatorial^1,2^. However, the classical strategies of drug discovery from natural products have some obstacles, such as the high complexity of crude extracts that could slow down the isolation of active metabolites. Additionally, in many cases, the bioactive metabolites could be found in small or trace quantities and masked by less active major ones. Additionally, the applied analytical method could identify a small fraction of the active components in the given extracts^3^. Therefore, several previous studies have reported that the isolated pure compounds were less pharmacologically active than their corresponding crude extracts e.g. *Artemisia annua* crude extract was more active as an antimalarial agent than its marker pure metabolite, artemisinin (**11**)^4^.

Metabolomics has become an emerging strategy that can be utilized for the comprehensive characterization of complex crude extracts along with targeting marker metabolites that can be associated with certain biological activities before commencing time-consuming purification procedures^5–7^. The combination of suitable analytical tools (e.g. Nuclear Magnetic Resonance; NMR and Liquid Chromatograph coupled with High-Resolution Mass Spectrometry; LC-HRMS) with Multivariate Analysis (MVA) can profile a huge number of metabolites in a certain crude extract and determine their correlations with an observed pharmacological efficacy^8^. In case of targeting the isolation of novel compounds, LC-HRMS-based metabolomic profiling can dereplicate the known metabolites and highlight the probably new ones, and hence, save the time spent on the isolation and characterization of unwanted non-bioactive or known metabolites from a given extract^9,10^. The generated huge metabolomic data from a chosen analytical tool requires MVA for samples classification into different groups and to investigate the metabolites distribution among these groups^11^. Among the commonly used MVA, Principal Component Analysis (PCA), Partial Least Square-Discriminant Analysis (PLS-DA) and Orthogonal Partial Least Square-Discriminant Analysis (OPLS-DA) are the main tools used for this purpose^12^.

*Artemisia annua* L. (aka sweet wormwood) is a well-known antimalarial herb and considered the main source of artemisinin (**11**), the first-line antimalarial drug. According to the latest World Health Organization (WHO) malaria report, around 228 million malaria cases were reported in 2018 and led to 405,000 deaths. Additionally, it reported the spread of artemisinin-resistance among patients in the South East Asia region^13^. Despite, *A. annua* has been comprehensively investigated as a crucial source of potent antimalarial agents, its associated fungal endophytes could offer further new ones.

There are many examples on fungal endophytes that have reported to produces bioactive metabolites similar to those originally derived from the host plant. The best known example is the discovery of the paclitaxel -producing endophytic fungus *Taxomyces andreanae* that had been isolated from the pacific yew *Taxus brevifolia*. Vincristine is also another anticancer drug originally reported from *Catharanthus roseus*, was later detected in cultures of its endophytic fungus, *Fusarium oxysporum.* Recently, we have investigated the endophytes associated with the medicinal plant *Solanum nigrum*, and found one of them (i.e. Aspergillus flavus) able to produce the solamargine which is considered one of the characteristic metabolites in this plant^14^.

**Figure 1:**
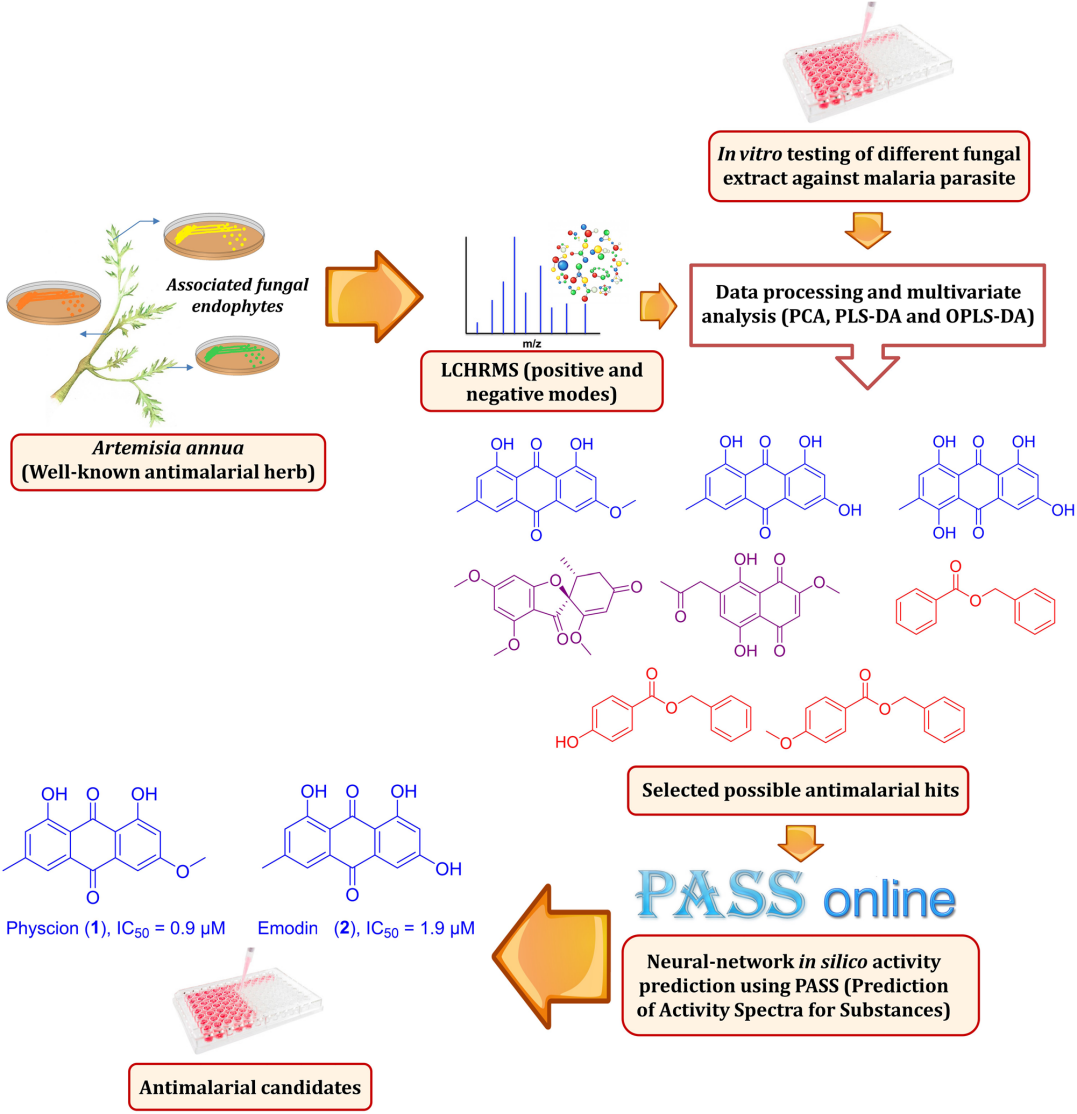
The applied metabolomics and in silico screening strategies in the present study.

Consequently, we aimed in this study to investigate the fungal endophytes associated with the antimalarial herb *A. annua* and reveal their possible anti-plasmodial potential. Using an untargeted metabolomics approach, metabolites that may have in vitro anti-plasmodial activity can be systematically detected and identified directly from their corresponding fungal crude extracts. Subsequently, a neural-networking-based in silico prediction was applied to further support the metabolomic analysis predictions and inspect the highest possible active compounds and their potential target proteins, so that they could be subjected to in vitro validation. The applied strategy in the present study is depicted in Fig. 1.

## Results and discussion

### Identity and phylogeny of isolated fungal endophytes

**Figure 2:**
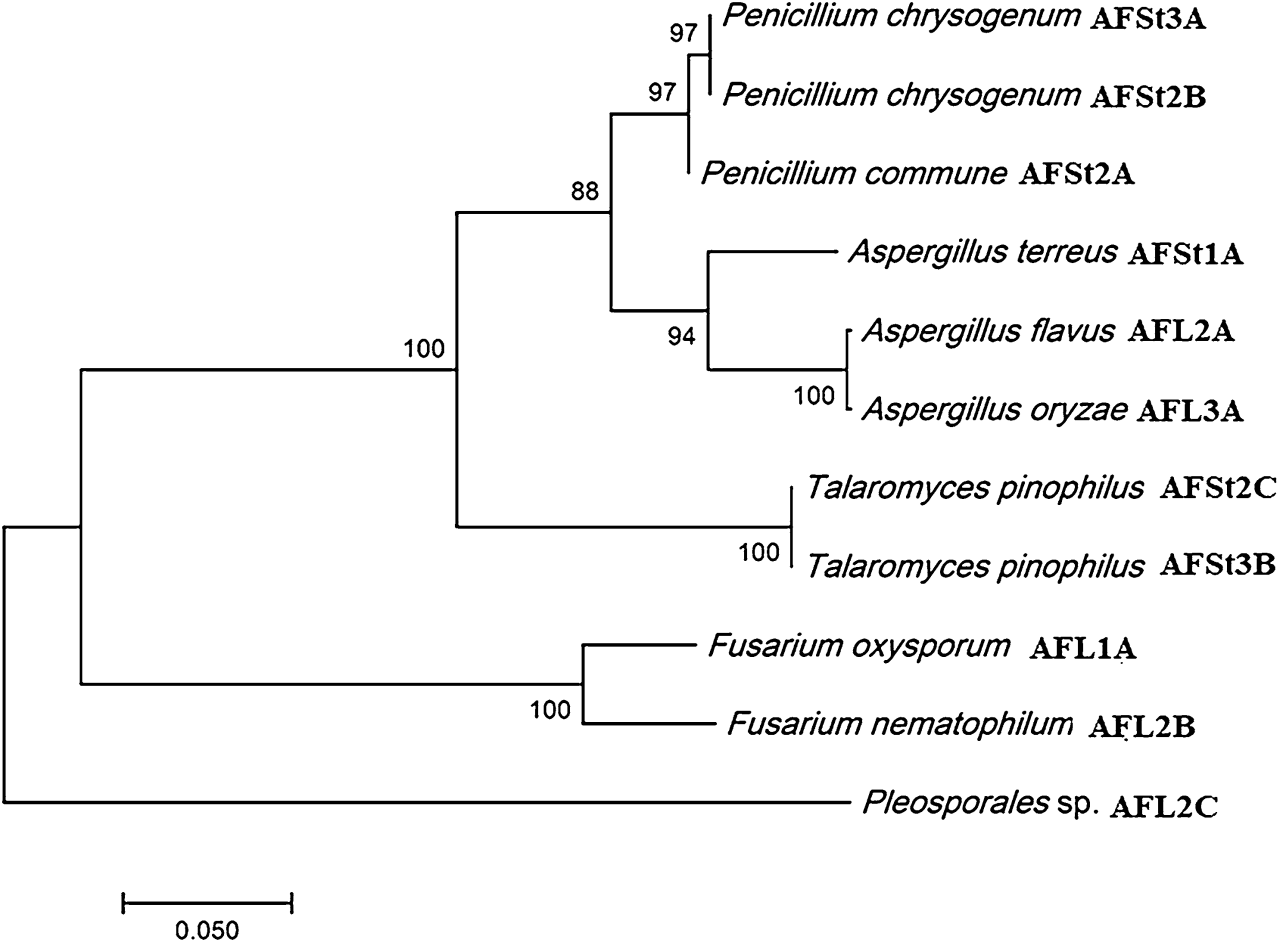
Neighbour‐joining phylogenetic tree based on ITS‐rDNA sequences of *A. annua*-derived fungal endophytes.

In total, eleven endophytic fungal isolates were recovered from healthy above-ground tissue (leaf, stem) of three plant specimens (*A. annua*) (Table 1). The amplified ITS region of the fungal strains was sequenced and compared with the ITS sequences of microorganisms represented in the NCBI database GenBank using blast search and MEGA 7 (http://www.megasoftware.net/) to generate a phylogenetic tree (Fig. 2) with the method of Neighbour-joining tree algorithm and the evolutionary distances were figured using the Kimura 2-parameter method. Eight strains were found to represent the Trichocomaceae family and belong to the genera *Aspergillus* (three strains), *Penicillium* (three strains), *Talaromyces* (two strains), two *Nectriaceae* representatives belonging to the genera *Fusarium*, and only one representative of family Pleosporaceae. The resulted nucleotide sequences were deposited in GenBank under accession numbers (Table 1).

**Table 1:** *A. annua*-derived fungal endophytes and their GenBank accession numbers, and the in vitro anti-plasmodial activity of their corresponding extracts.

No	Isolate	Fungal ID	Accession number	Antiplasmodial activity (IC_50_, µg/mL)
1	AFSt1A	*Aspergillus terreus*	MT300182	35 ± 1.1
2	AFL2A	*Aspergillus flavus*	MT300178	> 50
3	AFL3A	*Aspergillus oryzae*	MT300184	> 50
4	AFSt2A	*Penicillium commune*	MT300172	1.1 ± 1.7
5	AFSt2B	*Penicillium chrysogenum*	MT300171	2.1 ± 1.4
6	AFSt3A	*Penicillium chrysogenum*	MT300180	3.3 ± 2.1
7	AFSt2C	*Talaromyces piophilus*	MT300175	7.6 ± 2.4
8	AFSt3B	*Talaromyces piophilus*	MT300176	9.9 ± 2.1
9	AFL1A	*Fusarium oxysporum*	MT300173	> 50
10	AFL2B	*Fusarium nematophilum*	MT300179	> 50
11	AFL2C	*Pleosporaceae* sp.	MT300181	> 50

### In vitro anti-plasmodial activity

All isolated fungal endophytes were cultivated on malt extract media (ME), and their corresponding ethyl acetate extracts were prepared. ME was the culture medium of choice, based on our previous experiments on other fungal endophytes^14,15^. Subsequently, all prepared extracts were screened for their in vitro anti-plasmodial activity using the pathogenic strain *Plasmodium falciparum*. The extracts derived from the three *Penicillium* strains revealed the highest inhibitory activity with IC_50_ values ranged from 1.1 to 3.3 µg/mL, followed by the extracts of *Talaromyces* strains (IC_50_ 7.6 ± 2.4, 9.9 ± 2.1 µg/mL) and finally *Aspergillus terreus*-derived extract was the least active one (IC_50_ 35 ± 1.1 µg/mL) (Table 1). Interestingly, all extracts that showed anti-plasmodial activity were prepared from the fungal endophytes of the plant’s stem, and hence, such observation need further future investigation to find out the probable link between the endophytes’ inhabitant place and their biological activity.

### Metabolomic analysis

#### LC-HRMS chemical profiling of the extracts

LC-HRMS analysis of the fungal extracts demonstrated a huge diversity of secondary metabolites with a total of 2363 peaks were detected in the eleven endophytic fungi under study. Since the metabolites profile in each extract is varying in their physical nature and ionization potential, both the positive and negative ionization modes were applied so that detection of the maximum possible metabolites was accomplished^16^ (Supplementary material S1). To correlate between the in vitro anti-plasmodial activity (Table 1) and the possible metabolites responsible for this observed activity, an MVA on the generated LC-HRMS data needs to be performed. Subsequent in silico calculations would support the MVA prediction, and hence prioritize metabolites that are most likely associated with the in vitro inhibitory activity.

#### Data interpretation and multivariate analysis (MVA)

**Figure 3:**
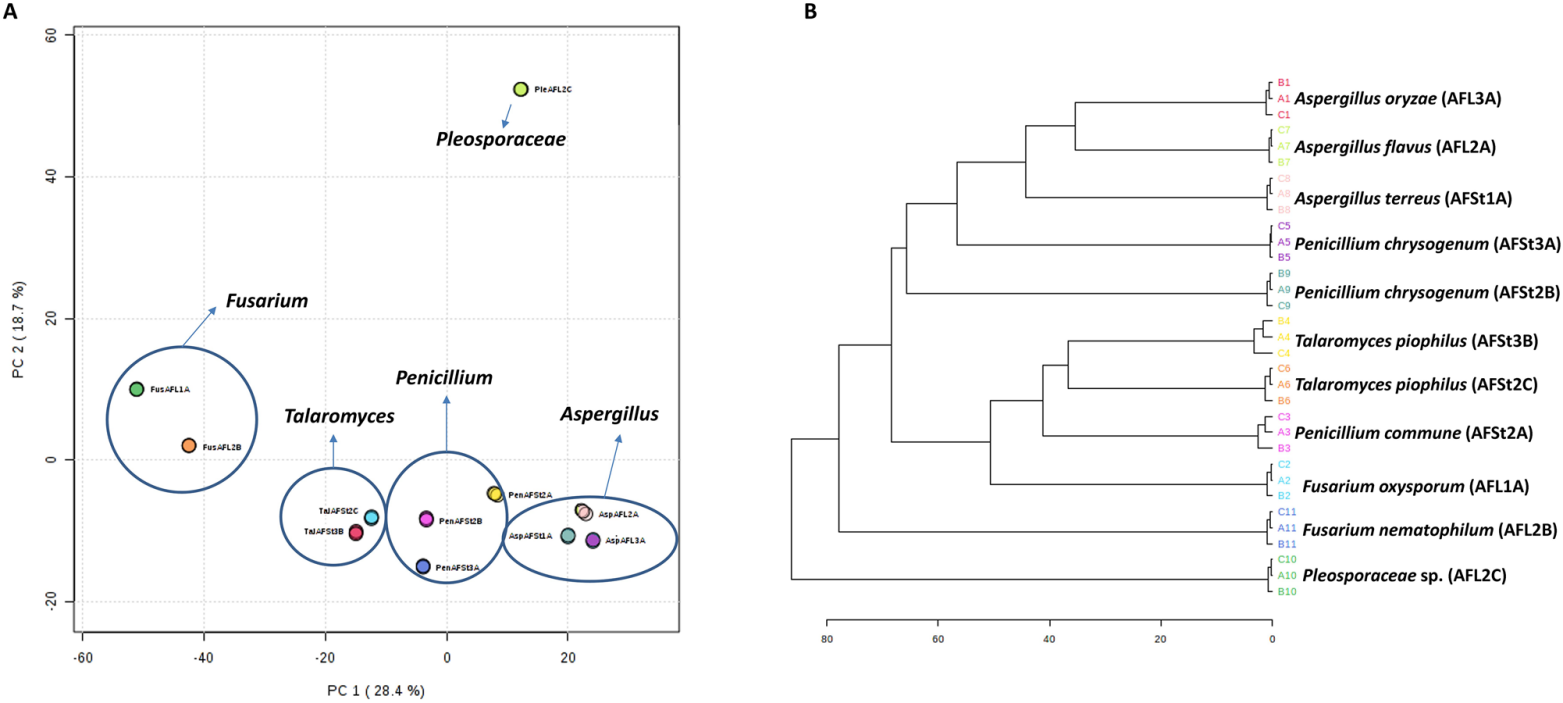
Score plots representing PCA (**A**) based on the HRMS data obtained for the endophytes extracts, and their chemotaxonomic clustering dendrogram (**B**). The plots were generated by MetaboAnalyst 4.0^28^ (https://www.metaboanalyst.ca/MetaboAnalyst/ModuleView.xhtml).

PCA score plot of the HRMS data (Fig. 3A) showed the clustering of the endophyte-derived extracts according to their phylogenetic relationship. Hierarchical Cluster Analysis (HCA) derived from the PCA (Fig. 3B) results revealed that extracts prepared from the same fungal genera were grouped. Moreover, HCA dendrogram illustrated that endophytes belong to *Aspergillus*, *Penicillium*, and *Talaromyces* genera were close to each other, and significantly separated from those of *Fusarium* and *Pleosporaceae*, similar to their phylogenetic analysis (Fig. 2). This indicated that the metabolomic analysis of a group of related organisms can be used as a chemotaxonomic tool along with phylogenetic proximity analysis.

#### Metabolites-bioactivity relationship

**Figure 4:**
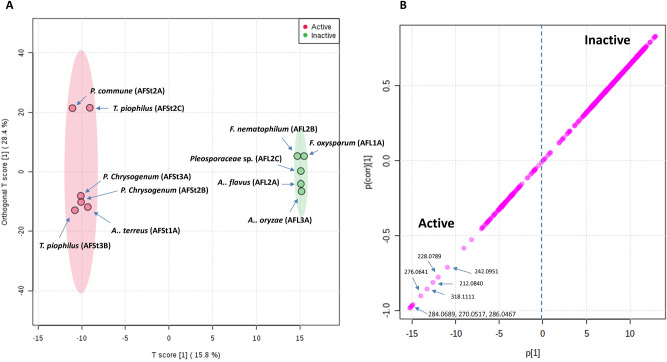
OPLS-DA score plot (**A**) along with its S-plot illustrating the masses of the most important metabolites (**1–8**) that may be associated with the anti-plasmodial activity (**B**). The plots were generated by MetaboAnalyst 4.0^28^ (https://www.metaboanalyst.ca/MetaboAnalyst/ModuleView.xhtml).

OPLS-DA was applied to explore the relationship between the observed in vitro anti-plasmodial activity of the endophyte-derived extracts and their metabolites profiles. The generated model showed good performance (goodness of models, *R*^2^ = 0.89) and prediction (predictive power of models, *Q*^2^ = 0.9). *R*^2^ values very close to 1.0 were the best, although values > 0.5 were also considered good due to the chemical complexity of the tested samples^11,17^. Extracts with IC_50_ values ≤ 50 µg/mL were designated as active anti-plasmodial and the remaining values as inactive. OPLS-DA-derived score plot (Fig. 4A) showed clear separations between active and inactive extracts. Additionally, active extracts were closely clustered together indicating that there were a group of metabolites in these active extracts that could be responsible for the observed anti-plasmodial activity. The OPLS-DA-derived S-Plots (Fig. 4B) were used to predict the bioactive discriminating marker metabolites that were linked to the observed antimalarial activity of the active extracts (Table 2).

**Table 2:** Metabolites correlated to the anti-plasmodial activity of the tested endophyte-derived extracts.

No	Rt	*m/z*	Ionization mode	Accurate mass	Calculated mass	Molecular formula	MS^2^	Putative identification	Chemical class
1	9.52	285.0762	Positive	284.0689	284.0685	C_16_H_12_O_5_	270.0537, 242.0578	Physcion^a^	Anthraquinone
2	7.78	269.0451	Negative	270.0517	270.0528	C_15_H_10_O_5_	241.0514, 225.0569	Emodin^a^	Anthraquinone
3	5.27	285.0395	Negative	286.0467	286.0477	C_15_H_10_O_6_	257.0454, 243.0295, 173.0244	Katenarin	Anthraquinone
4	4.51	277.0714	Positive	276.0641	276.0634	C_14_H_12_O_6_	259.0630, 245.0442, 221.0454, 193.0499	Norjavanicin	Quinone
5	4.57	319.1185	Positive	318.1111	318.1103	C_17_H_18_O_6_	301.1081, 259.0611, 219.0656	Dechlorogriseofulvin	Polyketide
6	8.71	213.0911	Positive	212.0840	212.0837	C_14_H_12_O_2_	197.0606, 187.0757, 185.0607	Benzyl benzoate^a^	Aromatic ester
7	5.55	229.0868	Positive	228.0789	228.0786	C_14_H_12_O_3_	211.0757, 199.0754, 187.0757	4-Hydroxy benzyl benzoate^a^	Aromatic ester
8	8.85	243.1025	Positive	242.0951	242.0943	C_15_H_14_O_3_	227.0712, 213.0913, 197.0605, 187.0755, 150.9683	Benzyl anisate^a^	Aromatic ester

^a^Its identity was further confirmed by comparison with authentic standards.

**Figure 5:**
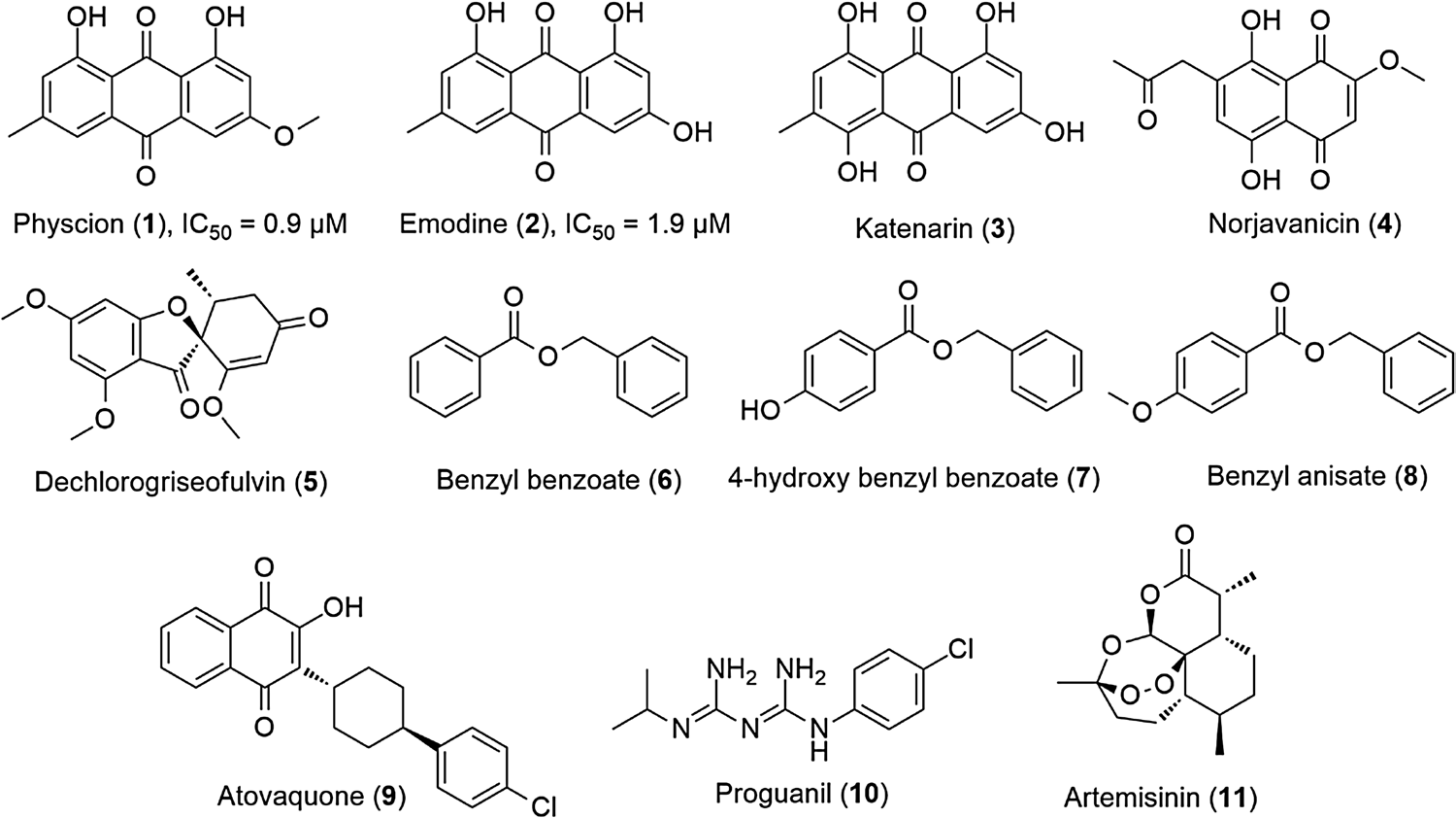
Structures of metabolites (**1–8**) that were highly correlated with the extracts’ anti-malarial activity alongside other well-known antimalarial agents (**9–11**).

A total of eight metabolites (**1–8**) (Table 2, Fig. 5) were tentatively characterized depending on the comparison with authentic standards, and assessment of their MS/MS fragmentation patterns (Supplementary material S1) with those reported in the literature, the MassBank (MoNA; https://mona.fiehnlab.ucdavis.edu/) and the Competitive Fragmentation Modeling for Metabolite server (CFM-ID 3, http://cfmid.wishartlab.com/).

Quinone derivatives (**1–4**) were found to be the main metabolites strongly correlated to the anti-plasmodial activity. In a previous report, a number of quinones,particularly anthraquinones, have also revealed a promising antimalarial potential^18^. Moreover, atovaquone (**9**) is a well-known antimalarial quinone derivative that is used in combination with proguanil (**10**) (MALARONE) for the management of malaria infections worldwide. Regarding dechlorogriseofulvin (**5**), we could not find any previous studies dealing with its possible anti-plasmodial activity. Furthermore, we were able to characterize aromatic ester derivatives namely benzyl benzoate (**6**), 4-hydroxy benzyl benzoate (**7**) and benzyl anisate (**8**), which were also linked to the extract’s anti-plasmodial effect. Compound (**6**) was reported as a major component in *Cinnamomum zeylanicum*^19^ and shown to exert a potent scabicidal activity^20^.

**Figure 6:**
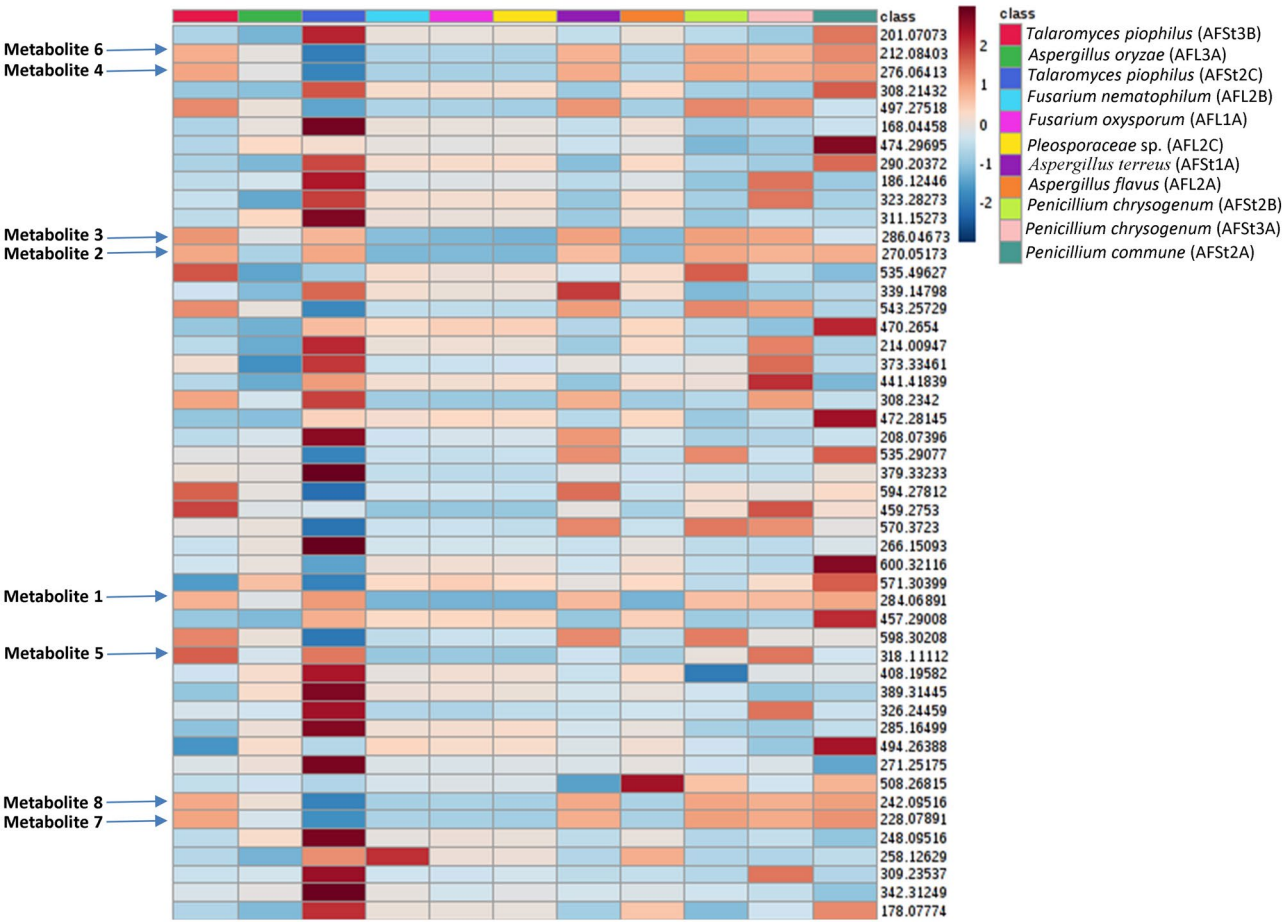
Heat-map indicating the distribution and abundance of main metabolites in the active antimalarial extracts including metabolites (**1–8**) that were highly correlated with this observed activity. The heat map was generated by MetaboAnalyst 4.0^28^ (https://www.metaboanalyst.ca/MetaboAnalyst/ModuleView.xhtml).

The heat map in Fig. 6 illustrated the distribution of these bioactivity-linked metabolites among the endophytes under study. Based on previous findings, we would argue that the utilization of sensitive analytical techniques together with a proper MVA could facilitate targeting the biomarkers of specific biological activity.

### In silico predictions and in-vitro validation

**Figure 7:**
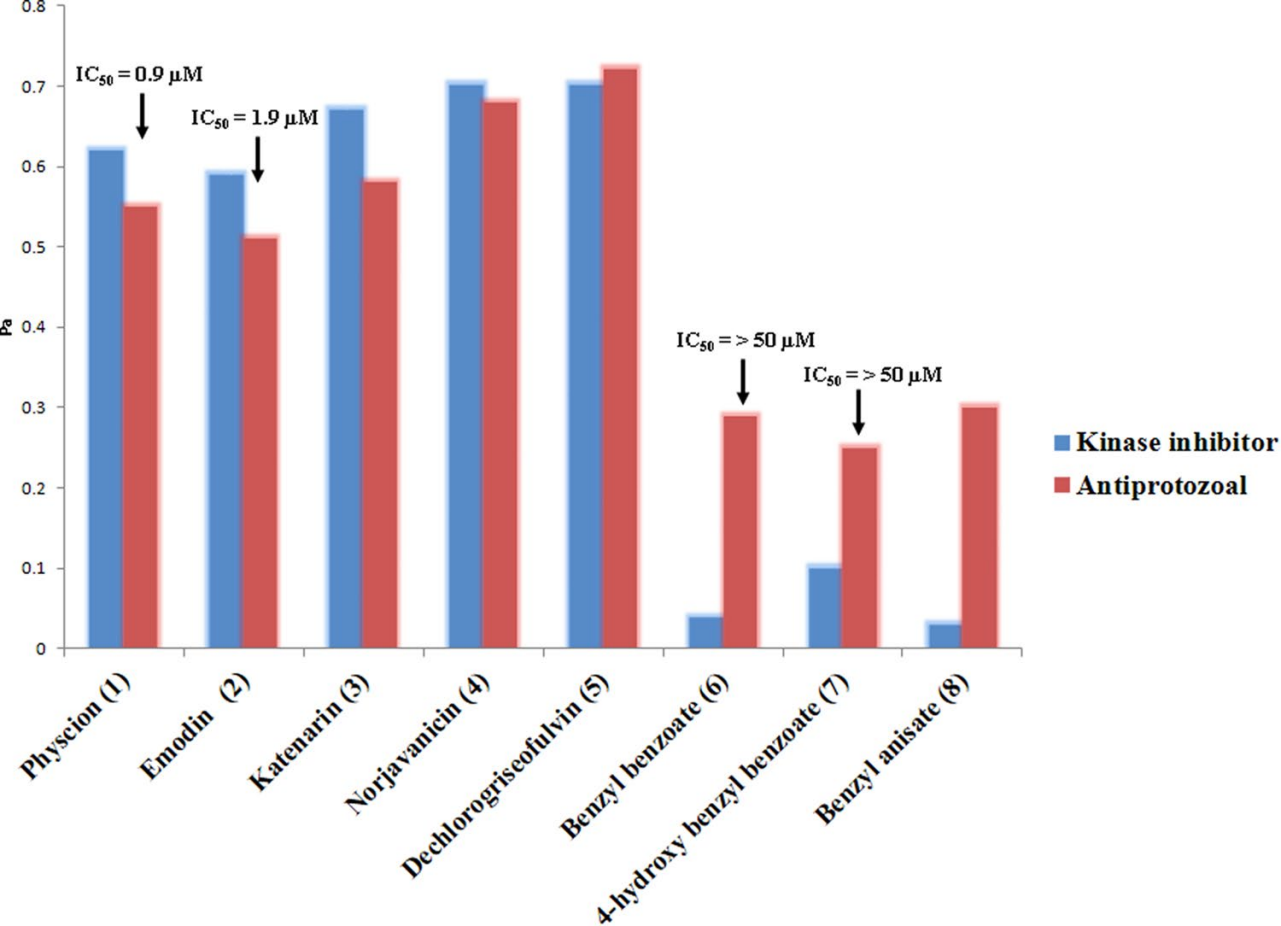
PASS prediction scores of metabolites (**1–8**) as possible antiprotozoal and kinase inhibitors. Pa scores > 0.5 indicated high possible experimental activity, while Pa < 0.5 indicated low possible experimental activity. Compounds **1** and **2** showed potent in vitro activity, while compounds **6** and **7** were in active.

To support the MVA prediction of the anti-plasmodial activity-linked metabolites, we further subjected these selected metabolites to a neural network-based software called Prediction of Activity Spectra for Substances (PASS). Interestingly, OPLS-DA-suggested compounds (**1–8**) were corroborated to the PASS prediction as antiprotozoal agents (malaria) with significant possible activity (Pa) scores of 0.25–0.72. Additionally, quinone-derived compounds (**1–5**) were also predicted to be possible kinase inhibitors candidates with Pa scores correlated to their predicted antimalarial activity (Pa: 0.51–0.72) (Fig. 7). These findings were supported by earlier reports that suggested the plasmodial kinase disruption as a possible target of anthraquinone-related compounds^21^. Regarding compounds (**6–8**), they were probably inactive as kinase inhibitors (Pa: 0.03–0.1) and had weak antimalarial activity (Pa: 0.25–0.3). To validate our MVA and PASS predictions, we selected representatives from compounds (**1–8**) for in vitro anti-plasmodial testing. Interestingly, compounds **1** and **2** showed potent inhibitory activity with IC_50_ values of 0.9 and 1.9 µM, respectively. On the other hand, both compounds **6** and **7** were inactive (Fig. 7).

The predicted Absorption, Distribution, Metabolism, Excretion and Toxicity (ADMET) profiles of physcion (**1**) and emodin (**2**) were calculated using the online software SwissADME and preADMET. Generally, both compounds showed excellent drug-like properties, high oral absorption, high bioavailability, and moderate toxicity (Tables 3 and 4). Such predicted toxicity (e.g., the mutagenic and carcinogenic characteristics) of both compounds should be taken into consideration during their development as antimalarial therapeutic agents in order to minimize toxicity.

**Table 3:** Predicted ADME profiles of physcion (**1**) and emodin (**2**).

Metabolite	Lipinski^a^	GIT Absorption^b^	BBB^c^	Solubility^d^	CYP2D6^e^	Bioavailability score^f^
Physcion (**1**)	Yes	High	No	High	No	0.55
Emodin (**2**)	Yes	High	No	High	No	0.55

^a^Predicts if the compound has a drug-like properties (follows the Lipinski rule of five).

^b^Predicts the gastrointestinal absorption according to the white of the boiled egg.

^c^Predicts the ability of the compound to penetrate the blood–brain barrier (BBB) according to the yolk of the boiled egg.

^d^Predicts the solubility of each compound in water.

^e^Predicts the cytochrome P450 inhibition.

^f^Predicts the bioavailability score, where values > 0.5 indicate acceptable bioavailability.

**Table 4:** Predicted toxicity profile of physcion (**1**) and emodin (**2**).

Metabolite	Physcion (**1**)	Emodin (**2**)
Mutagenicity	Mutagen	Mutagen
Carcinogenicity (mouse)	Negative	Negative
Carcinogenicity (rat)	Positive	Positive
hERG inhibition (cardiotoxicity)	Medium risk	Medium risk

Neural networks and deep learning-dependent software have become an integral part of the drug discovery platform, notably prediction software that has proven to be able in reducing the time and efforts required for screening of huge libraries of chemical compounds to find out possible drug candidates. Such in silico tools could be employed in the drug discovery from natural sources, where they can prioritize several possibly active metabolites among a complex mixture of chemical compounds present in a given natural extract, and hence isolation and identification efforts will be directed only to the top-scoring candidates.

## Material and methods

### Chemicals and standards

HPLC grade acetonitrile, methanol and water (Thermo Fisher Scientific Inc., Dublin, Ireland) were used for LC-HRMS analysis. Additionally, authentic standards as 4-hydroxy benzyl benzoate, benzyl benzoate, emodin, physcion and ampicillin were obtained from Sigma-Aldrich Chemical Co. (Arklow, Co. Wicklow, Ireland). Other chemicals used in the present study were supplied from Sigma-Aldrich Chemical Co. (Ireland) as an analytical grade.

### Isolation of endophytic fungi

The plant material was collected from a cultivated field near Minia University (GPS coordinates N 28° 06′ 35.57″, E 30° 45′ 1.08″). A voucher specimen of the plant was kept in the herbarium of Faculty of Science, Minia University (voucher specimen code: AA-112). Collected fresh plant material was washed with running tap water to get rid of the attached soil particles. Subsequently, they were subjected to surface sterilization using ethanol for 1 min followed by 3% sodium hypochlorite solution for 3 min and finally a serial washing in sterilized double-distilled water^14,15^. Afterwards, each plant organ (e.g. leaves and stems) was aseptically cut into smaller pieces. Thereafter, they were placed in malt extract agar plates (LOBACHEMIE, Mumbai, India) supplemented with ampicillin (0.5 mg/mL) to inhibit the growth of associated bacterial endophytes. Finally, the plates were incubated at 28 °C ± 2. The emerged colonies were sub-cultured several times to obtain pure fungal isolates which were kept at 4 °C (voucher specimen codes: AAF-101 to AAF-111).

### Molecular identification and phylogenetic analysis

All isolated fungal strains were taxonomically characterized by the extraction of their genomic DNA materials followed by PCR amplification and sequencing of the fungal internal transcribed spacer (ITS) region using the universal primers ITS1 and ITS4^14,15^. The Blast tool in National Center for Biotechnology Information (NCBI) was used to compare the good quality sequences to the GenBank database to identify the closest related species with highly similar sequences to the amplified ones. Finally, the multiple sequence alignment and phylogenetic analysis were accomplished using MEGA7 software^22^.

### Fermentation and preparation of extracts

The pure isolated fungal endophytes were fermented in 1.5 L malt extract liquid medium by placing 2 × 2 cm segments of fresh growing agar culture using 5 L Erlenmeyer flasks. The flasks were incubated at 20 °C ± 2 under static conditions for four weeks. At the end of fermentation, 300 mL of ethyl acetate was added to each flask to stop fermentation. The fungal mycelia alongside with the culture broth were subjected to ultrasound-assisted extraction with ethyl acetate (3 × 300 mL). The extracts were then evaporated using a rotary evaporator (IKA, Frankfurt, Germany).

### In vitro anti-plasmodial activity

To determine the anti-plasmodial effect of the fungal extracts on *P. falciparum* erythrocytic replication in vitro, the Malstat assay was used as described^23–25^. To synchronize the NF54 culture, parasites with many ring stages were centrifuged, the pellet was resuspended in five times pellet volume of 5% w/v sorbitol /ddH20 and incubated for 10 min at room temperature. Cells were washed once with RPMI to remove sorbitol and further cultivated^23^. Synchronized ring-stage parasites with 1% parasitaemia of *P. falciparum* NF54 strains were plated in triplicate in 96-well plates (200 µL/well) in the presence of a serial dilution of extracts dissolved in 0.5% v/v dimethyl sulfoxide (DMSO). The parasites were incubated with the extracts for 72 h at 37 °C in the presence of nitrogen-containing 5% O_2_ and 5% CO_2_. The incubation of parasites with DMSO at a concentration of 0.5% alone was used as negative control and 20% was used as positive control Afterwards, 20 µL was removed and added to 100 µL of the Malstat reagent (1% Triton X-100, 10 mg of l-lactate, 3.3 mg Tris and 0.33 mg of APAD (3-Acetylpyridine adenine dinucleotide) dissolved in 1 mL of distilled water, pH 9.0) in a new 96-well microtiter plate. The plasmodial lactate dehydrogenase activity was then assessed by adding a 20 μL mixture of NBT (Nitro Blue Tetrazolium)/Diaphorase (1:1; 1 mg/mL stock each) to the Malstat reaction. The optical densities were measured at 630 nm and the IC_50_ values were calculated from variable-slope sigmoidal dose–response curves using the GraphPad Prism program version 5.

### LC-HRMS metabolomic analysis

Metabolomic profiling^26,27^ was performed on the crude fungal extracts on an Acquity Ultra Performance Liquid Chromatography system coupled to a Synapt G2 HDMS quadrupole time-of-flight hybrid mass spectrometer (Waters, Milford, CT, USA). Chromatographic separation was performed on a BEH C18 column (2.1 × 100 mm, 1.7 µm particle size; Waters, Milford, CT, USA) with a guard column (2.1 × 5 mm, 1.7 µm particle size) and a linear solvent gradient of 0–100% eluent B at a flow rate of 0.3 mL·min^−1^ over 6 min, using 0.1% formic acid in water (v/v) as solvent A and acetonitrile as solvent B. The injection volume was 2 µL and the column temperature was 40 °C. MS-convert software was used to convert the raw data into sliced positive and negative ionization files. Then, the obtained files were subjected to the data mining MZmine 2.10 software (Okinawa Institute of Science and Technology Graduate University, Japan) for deconvolution, peak picking, alignment, deisotoping, and formula prediction. Dictionary of Natural Products (DNP) 2018 database was used for the dereplication and identification of compounds.

### Statistical and multivariate analysis

LC-HRMS-derived data were subjected to multivariate analysis (MVA) using MetaboAnalyst software^28^. Principal component analysis (PCA), Partial least squares discriminant analysis (PLS-DA) and Orthogonal Projections to Latent Structures Discriminant Analysis (OPLS-DA) were done to determine the variations in the metabolite composition in the samples. The signal intensity of all variables was log10 transformed. All variables were scaled to the unit variance for PLS-DA derived from the LC-HRMS data sets.

### Biological activity predictions using (PASS) software

The neural network-based software Prediction of Activity Spectra for Substances (PASS)^29^ (www.way2drug.com) was used for further prioritization of the antimalarial activity of the suggested compounds (**1–8**). This software can predict > 4000 types of pharmacological and toxicological activities including their mechanism of action, with approximately 85% as acceptable precision, depending on the submitted compound structures that were subsequently screened utilizing the structure–activity relationship database (SARBase). The prediction results were expressed as probabilities scores (probably active “Pa” or probably inactive “Pi”). These calculated probability scores were determined by linking the structure and functional groups features in the tested molecules that matched or mismatched the specific activities listed in the software-associated database. The higher the Pa values, the more probable the compound to display the suggested pharmacological activity on a scale of 0–1. Pa values higher than 0.5 mean high experimental chance of the suggested pharmacological activity.

### In silico ADMET profiling

Drug-like properties and ADMET profiles of physcion (**1**) and emodin (**2**) were predicted using the online software SwissADME (http://www.swissadme.ch/) and PreADMET (https://preadmet.bmdrc.kr/adme/)^30^. Gastrointestinal (GIT) absorption, blood–brain barrier (BBB), solubility, bioavailability score, and inhibition of CYP2D6 were selected as ADME descriptors to be calculated, while carcinogenicity (rat and mouse), mutagenicity, and in vitro hERG inhibition (cardiotoxicity) were selected as toxicity descriptors.

### Statistical analysis

All results in the present study were obtained from three repeated biological experiments. The results were expressed as the means ± SEM of the indicated number of experiments (n ≥ 3). The statistical significance of differences between means was established by ANOVA with Duncan’s post hoc tests. *P* values < 0.05 were considered to indicate statistical significance.

## Conclusion

The present study investigated the diversity and antimalarial activity of the endophytic fungi associated with *A. annua* growing in Egypt. According to our results, the eleven isolated endophytic fungi were found to be members of five different genera; *Aspergillus*, *Penicillium*, *Talaromyces*, *Fusarium*, and *Pleoporaceae*. Similar to their host plant, all the isolated endophytic *Penicillium* and *Talaromyces* extracts exerted significant antimalarial activity. LC-HRMS-based metabolomics could provide sensitive and comprehensive chemical profiling of complex biological matrices and hence, application of such valuable analytical tools together with multivariate statistical analysis would assist in the taxonomical classification of the isolated endophytes according to their chemical profiles. Moreover, OPLS-DA was able to suggest the most probable metabolites associated with the antiplasmodial activity. Depending on their characteristic MS/MS and authentic comparison, these suggested metabolites were identified to be a group of quinone-derived compounds and aromatic ester derivatives. Neural-networking and deep learning in silico calculations in biomedical fields have become a powerful and integral tool in the prediction and modelling experiments. Using PASS software that utilizes advanced neural-networking and deep learning approaches, we found that some OPLS-DA-derived active metabolites (compounds **1–5**) were also classified as highly possible antimalarial agents. Furthermore, these metabolites were classified as kinase inhibitors based on previous reports on structurally related compounds. As a result of the previous extensive cheminformatic investigation, both physcion (**1**) and emodin (**2**) were validated this approach where they inhibited the in vitro plasmodial growth with IC_50_ values of 0.9 and 1.9 µM, respectively.

Similar biochemometrics approach has been extensively applied in natural products research as a preliminary evaluation step in the process of drug discovery from natural sources^31–34^. Hence, further integration with in silico approaches like neural network-based virtual screening that was applied in this investigation, can increase the success rate of targeting the bioactive metabolites in complex crude extracts.

## Supplementary Information

Supplementary Information

## Data Availability

The datasets generated and analyzed during the current study are available from the corresponding author on reasonable request.
