# Supplementary material for: A metabolomic approach to target antimalarial metabolites in the Artemisia annua fungal endophytes
Source: Sci Rep. 2021 Feb 2;11:2770. doi: 10.1038/s41598-021-82201-8 (PMC7854678; doi:10.1038/s41598-021-82201-8)
Supplement: Supplementary file 1 — Supplementary Information [file 41598_2021_82201_MOESM1_ESM.docx]

**Supplementary Material**

**A metabolomic approach to target antimalarial metabolites in the *Artemisia annua* fungal endophytes**

Hani A. Alhadrami^1,2§^**,** Ahmed M. Sayed**^3^**^§^, Ahmed O. El-Gendy**^4^**, Yara I. Shamikh**^5^**^,6^, Yasser Gaber^4,7,^ , Walid Bakeer^4^, Noheir H. Sheirf**^3,8^** Eman Zekry Attia**^9^**, Gehan Mohamed Shaban**^10^**, Basma Ali Khalifa**^10^**, Che Julius Ngwa^11^, Gabriele Pradel^11^, Mostafa E. Rateb ^12^, Hossam M. Hassan^13^, Usama Ramadan Abdelmohsen^9,14*^, Dalal Hussien M. Alkhalifah^15^, Wael N. Hozzein^*16,17^,

*^1^Department of Medical Laboratory Technology, Faculty of Applied Medical Sciences, King Abdulaziz University, Jeddah, 21589, Saudi Arabia;*

*^2^Special Infectious Agent Unit, King Fahd Medical Research Centre, King Abdulaziz University, P. O. Box 80402 Jeddah 21589, Saudi Arabia;*

*^3^Department of Pharmacognosy, Faculty of Pharmacy, Nahda University, Beni-Suef, Egypt 62513;*

*^4^Department of Microbiology, Faculty of Pharmacy, Beni-Suef University, Beni-Suef, Egypt 62514,*

*^5^Department of Microbiology and Immunology, Nahda University, Beni-Suef, Egypt 62513,*

*^6^Department of Virology, Egypt Center for Research and Regenerative Medicine (ECRRM), Egypt 11517,*

*^7^Department of Pharmaceutics and Pharmaceutical Technology, College of Pharmacy, Mutah University, Karak, 61710, Jordan;*

*^8^Drug Radiation Research Department, National Center for Radiation Research and Technology. Atomic Energy Authority;*

*^9^Department of Pharmacognosy, Faculty of Pharmacy, Minia University, Minia 61519, Egypt,*

*^10^Department of Botany and Microbiology, Faculty of Science, Minia University, Minia 61519, Egypt;*

*^11^Division of Cellular and Applied Infection Biology, Institute of Zoology, RWTH Aachen University, 52074 Aachen, Germany*

*^12^School of Computing, Engineering & Physical Sciences, University of the West of Scotland, Paisley PA1 2BE, UK;* [*mostafa.rateb@uws.ac.uk*](mailto:mostafa.rateb@uws.ac.uk)

*^13^Department of Pharmacognosy, Faculty of Pharmacy, Beni-Suef University, Beni-Suef, Egypt 62514;*

*^14^Department of Pharmacognosy, Faculty of Pharmacy, Deraya University, New Minia 61111, Egypt,*

*^15^Biology Department, College of Science, Princess Nourah Bint Abdulrahman University, Riyadh, Saudi Arabia;*

*^16^Bioproducts Research Chair, Zoology Department, College of Science, King Saud University, Riyadh, Saudi Arabia;*

*^17^Botany and Microbiology Department, Faculty of Science, Beni-Suef University, Beni-Suef, Egypt;*

*****Correspondence: [usama.ramadan@mu.edu.eg](mailto:usama.ramadan@mu.edu.eg) (URA) and hozzein29@yahoo.com (WNH);

**^§^**Equal contributions: Hani A. Alhadrami and Ahmed M. Sayed as first authors.


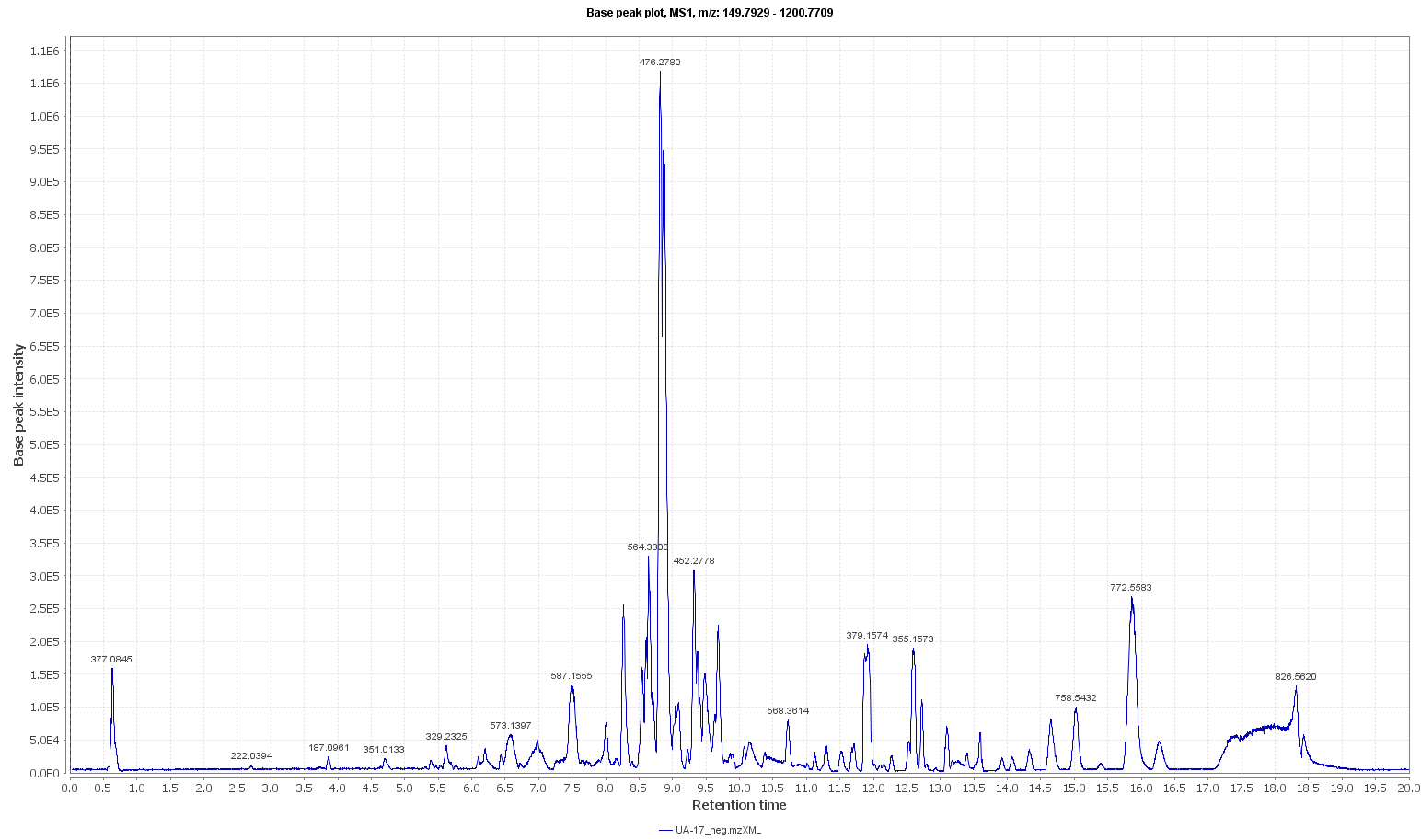


**Figure S1:** Total ion chromatogram of *Aspergillus terreus* (AFSt1A) extract.


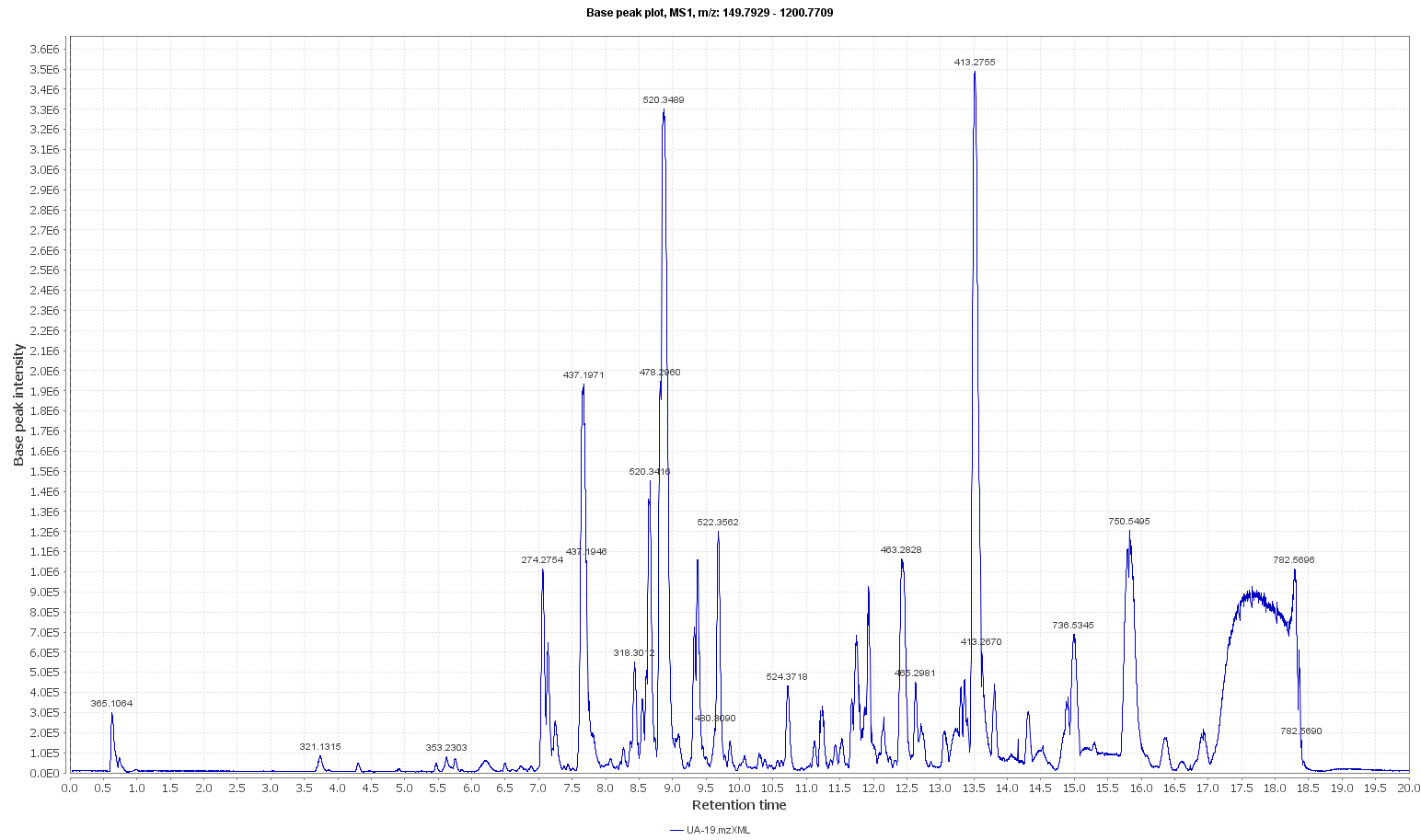


**Figure S2:** Total ion chromatogram of *Aspergillus flavus* (AFL2A) extract.


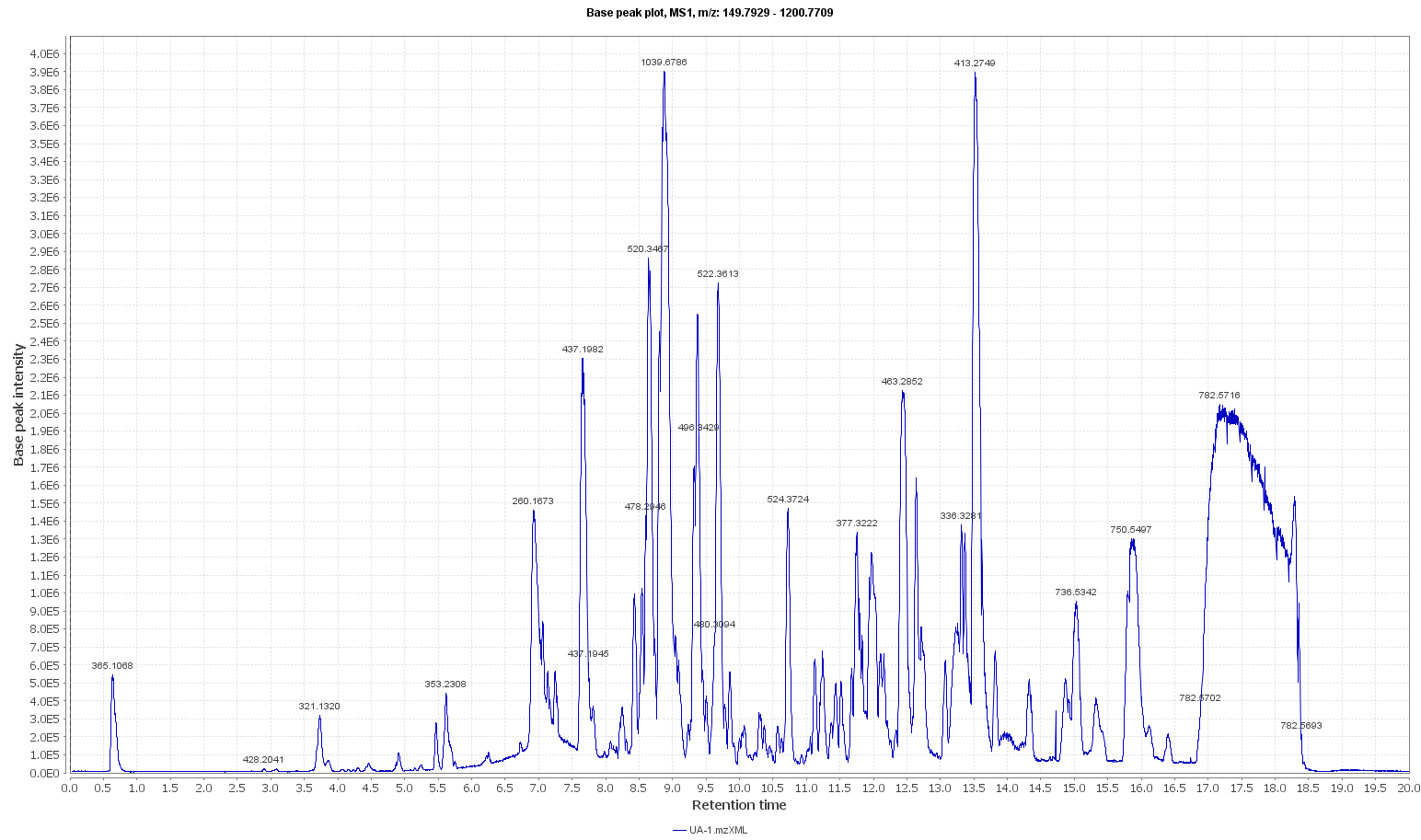


**Figure S3:** Total ion chromatogram of *Aspergillus oryzae* (AFL3A) extract.


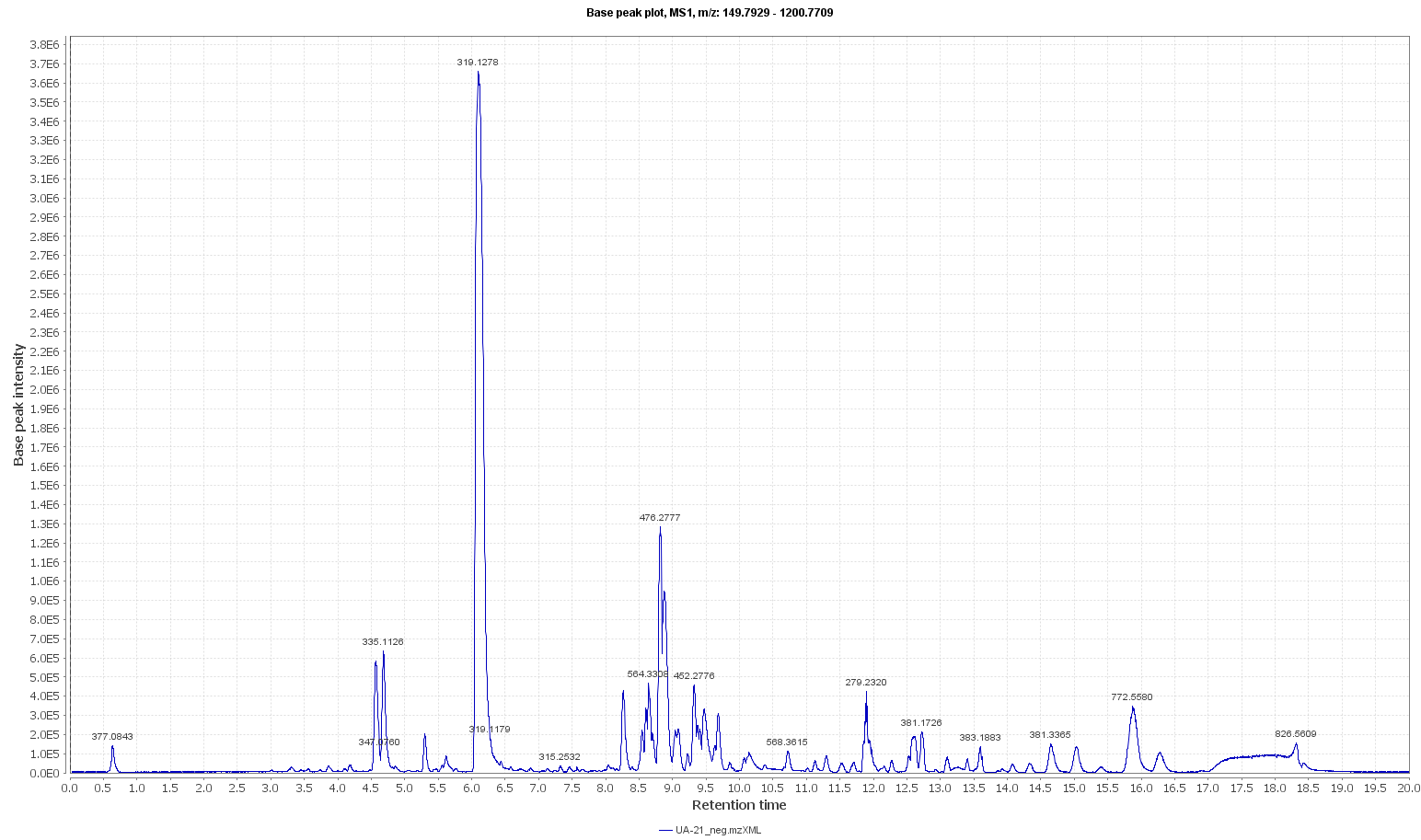


**Figure S4:** Total ion chromatogram of *Penicillium commune* (AFSt2A) extract.


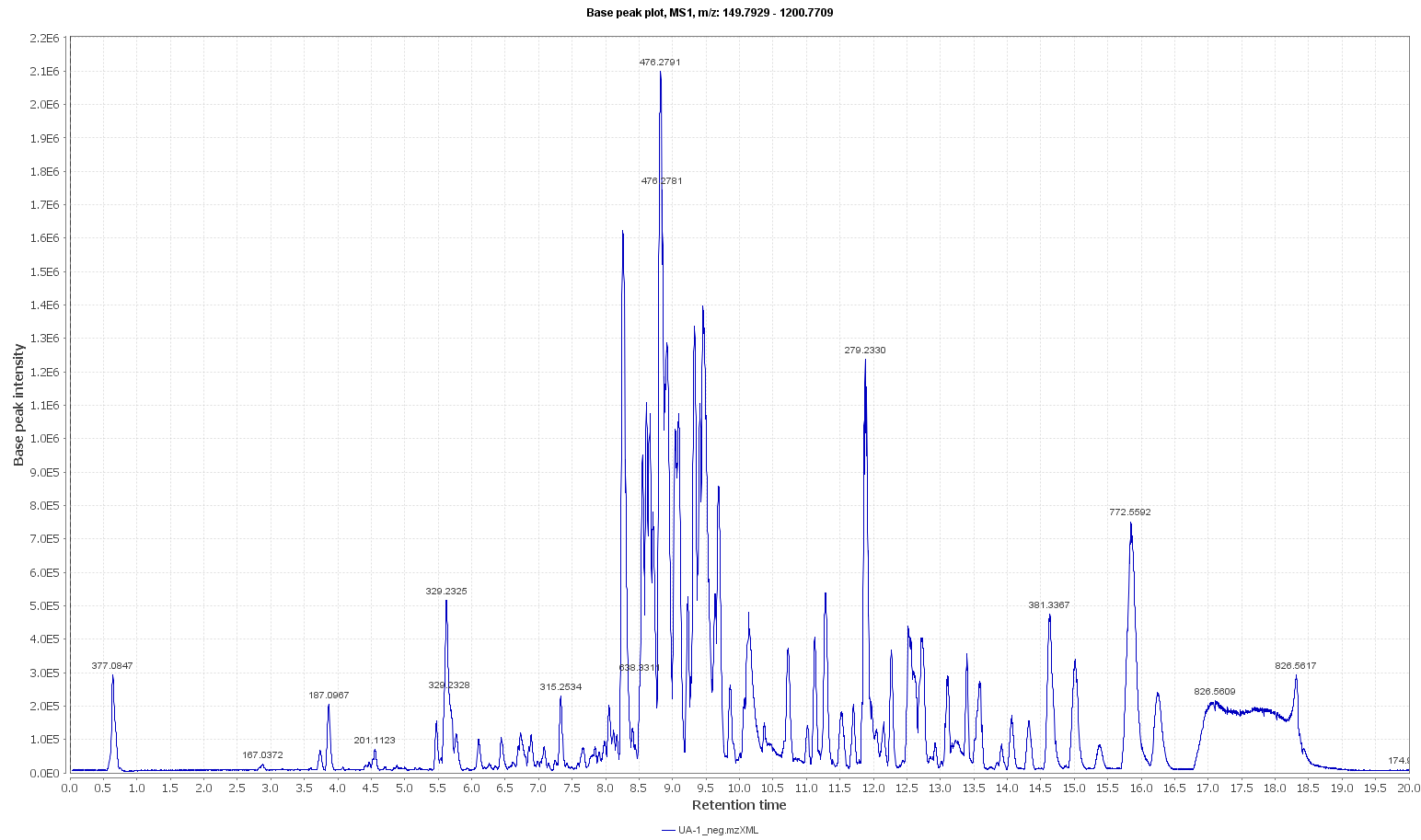


**Figure S5:** Total ion chromatogram of *Penicillium chrysogenum* (AFSt2B) extract.


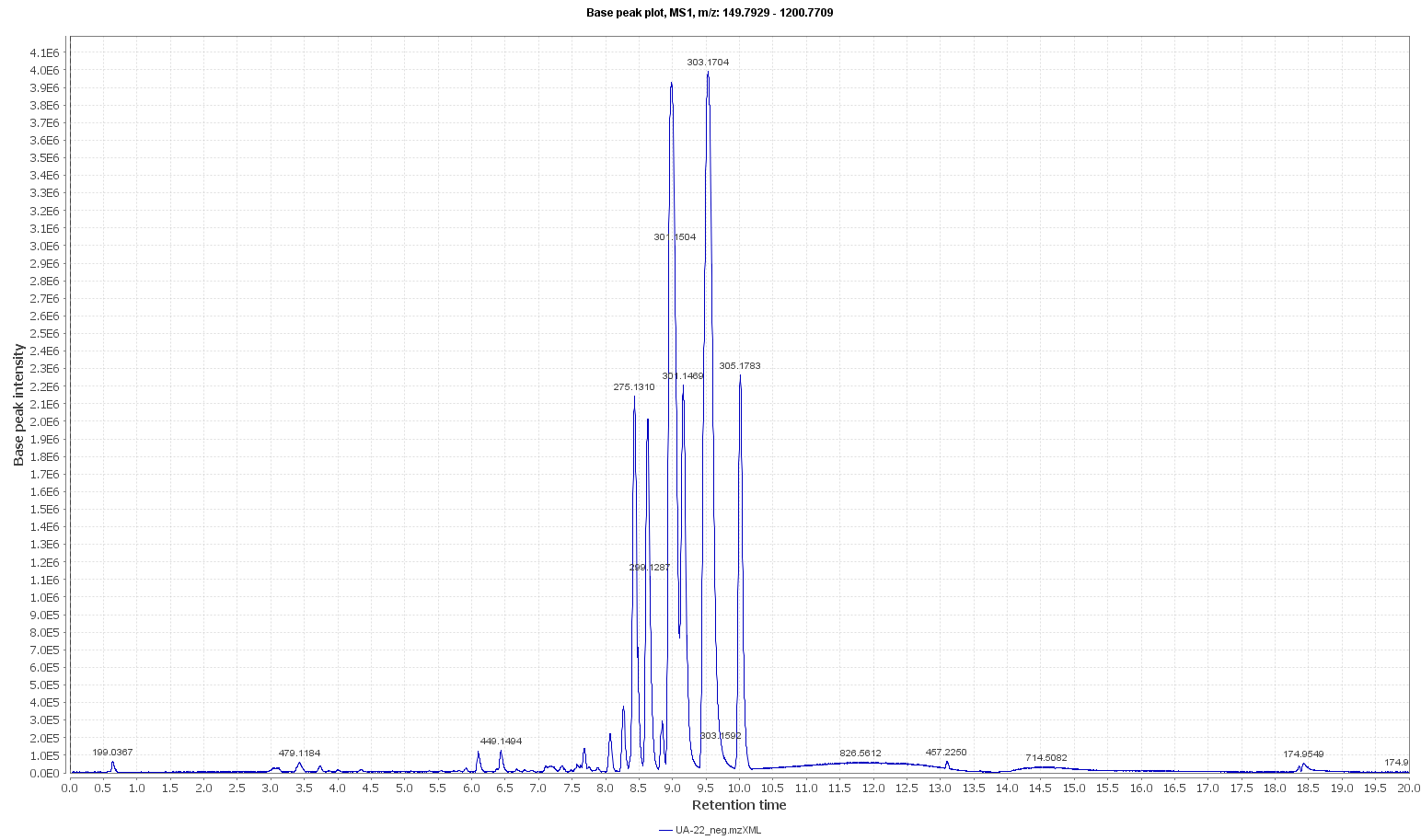


**Figure S6:** Total ion chromatogram of *Penicillium chrysogenum* (AFSt3A) extract.


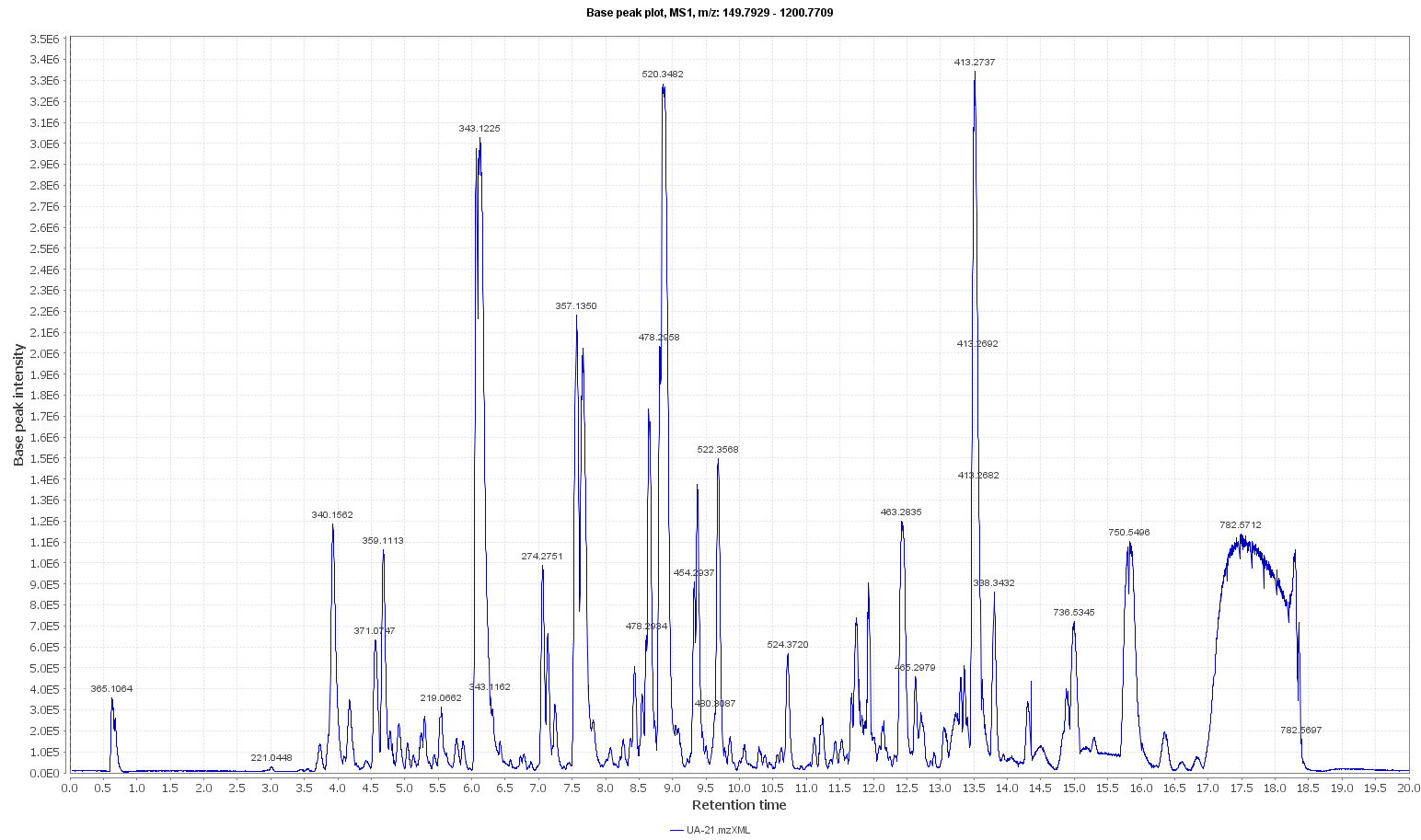


**Figure S7:** Total ion chromatogram of *Talaromyces piophilus* (AFSt2C) extract.


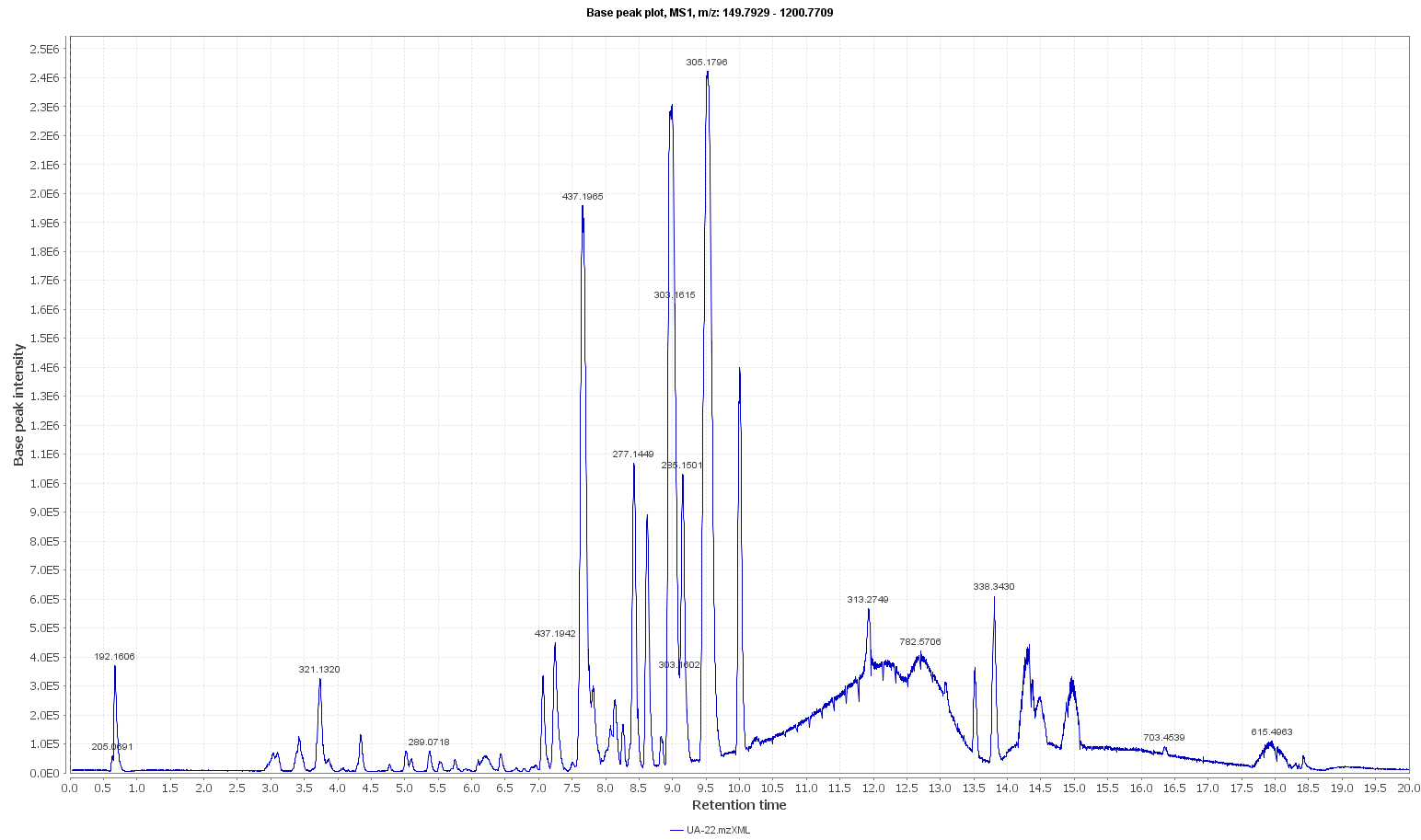


**Figure S8:** Total ion chromatogram of *Talaromyces piophilus* (AFSt3B) extract.


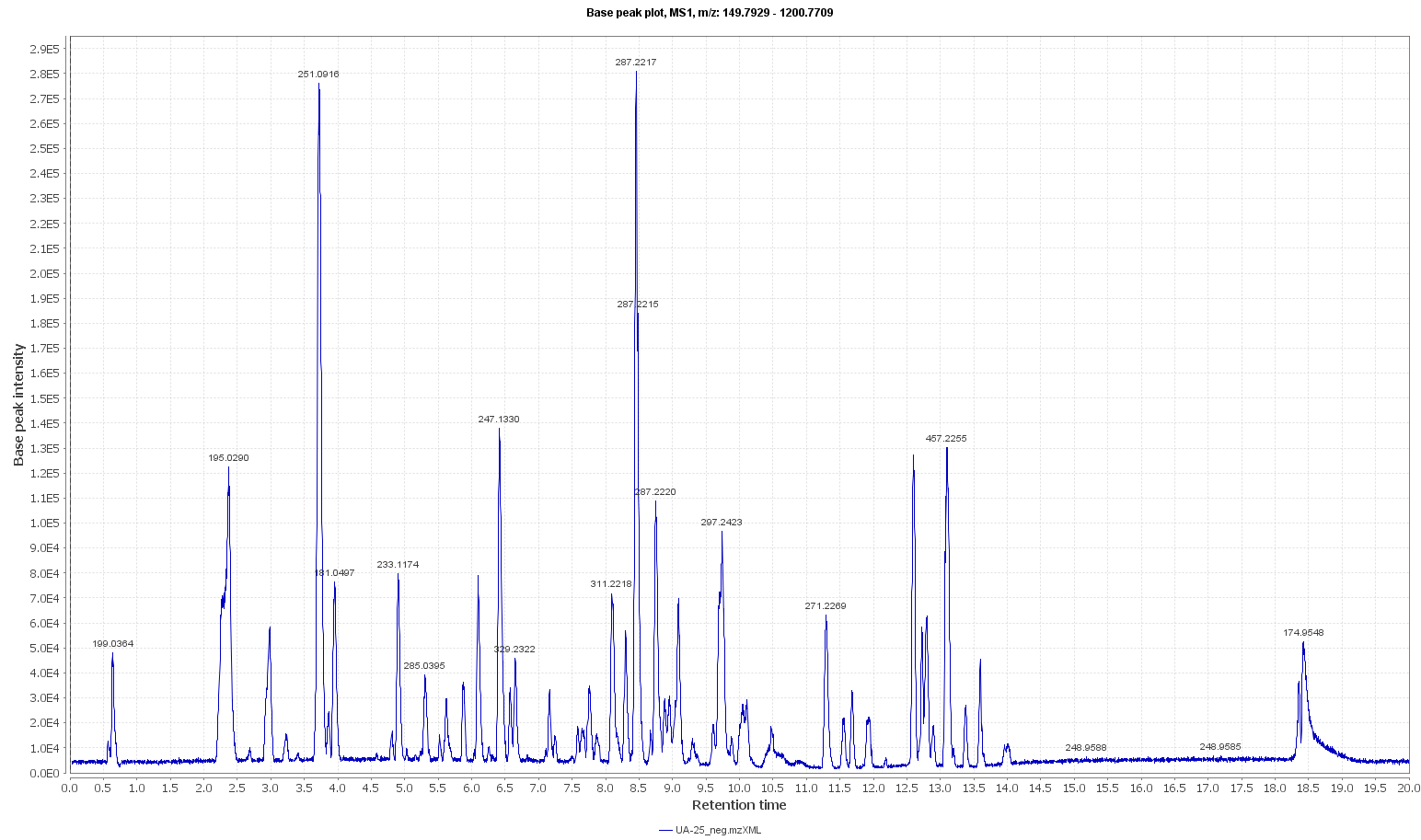


**Figure S9:** Total ion chromatogram of *Fusarium oxysporum* (AFL1A) extract.


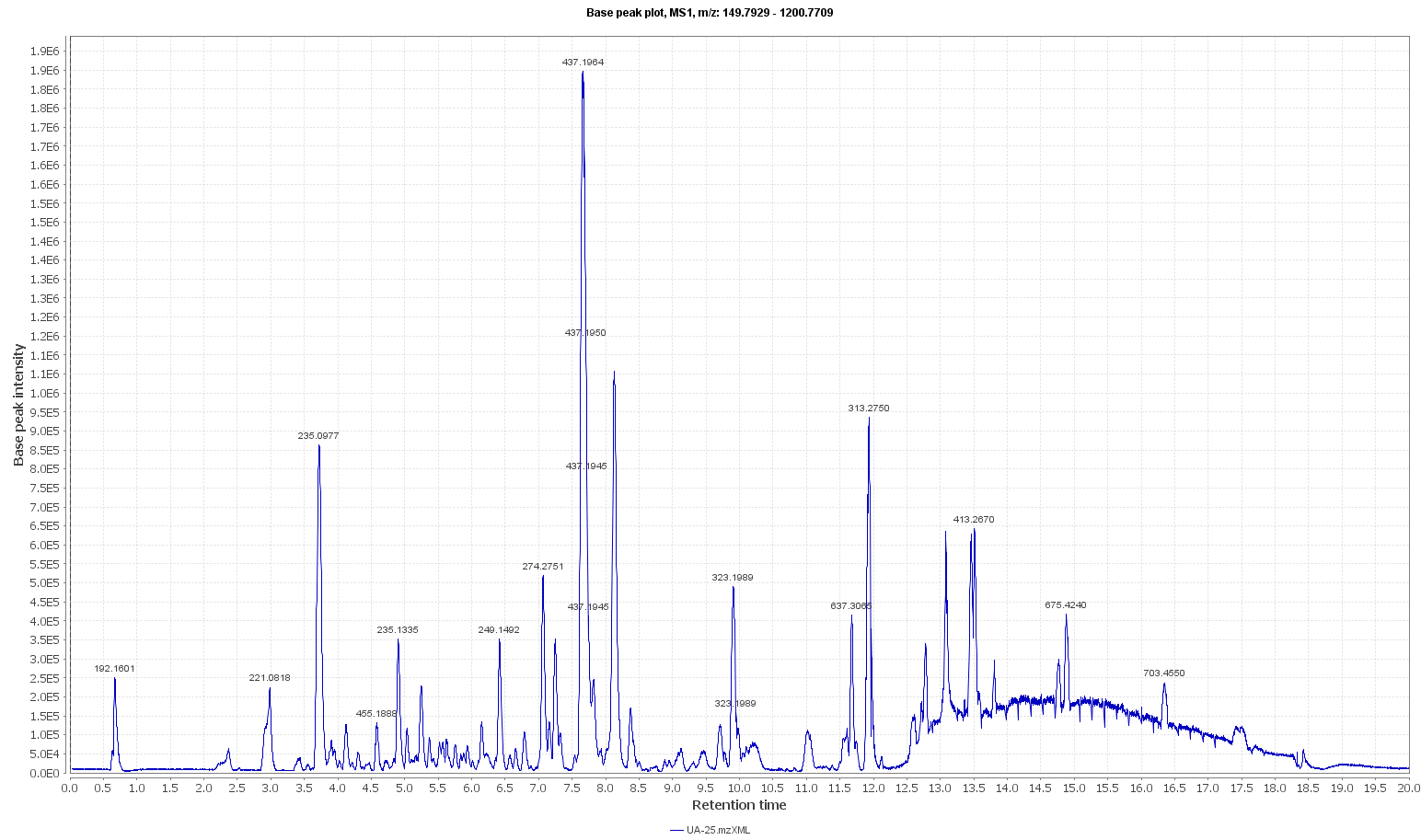


**Figure S10:** Total ion chromatogram of *Fusarium nematophilum* (AFL2B) extract.


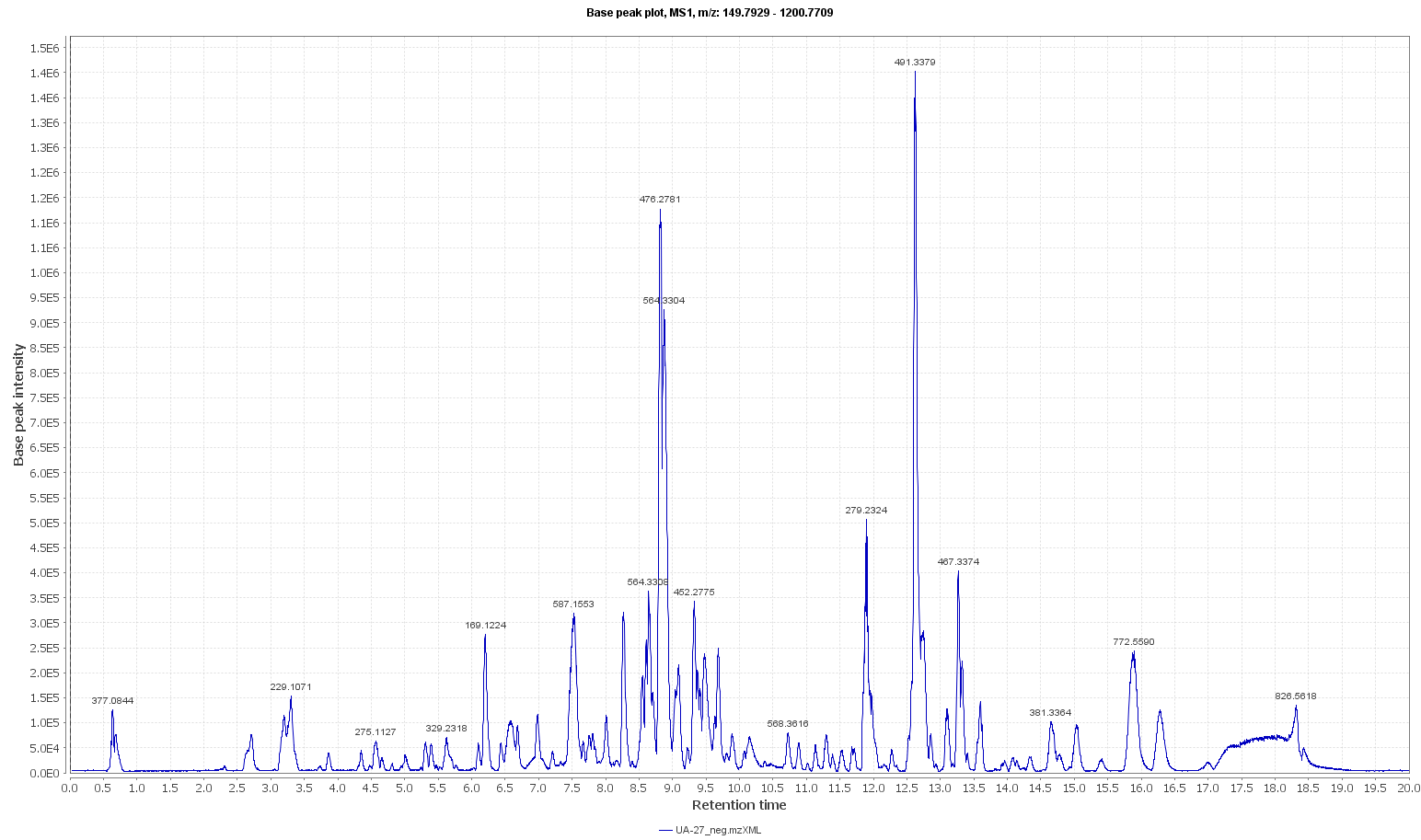


**Figure S11:** Total ion chromatogram of *Pleosporaceae* sp. (AFL2C) extract.


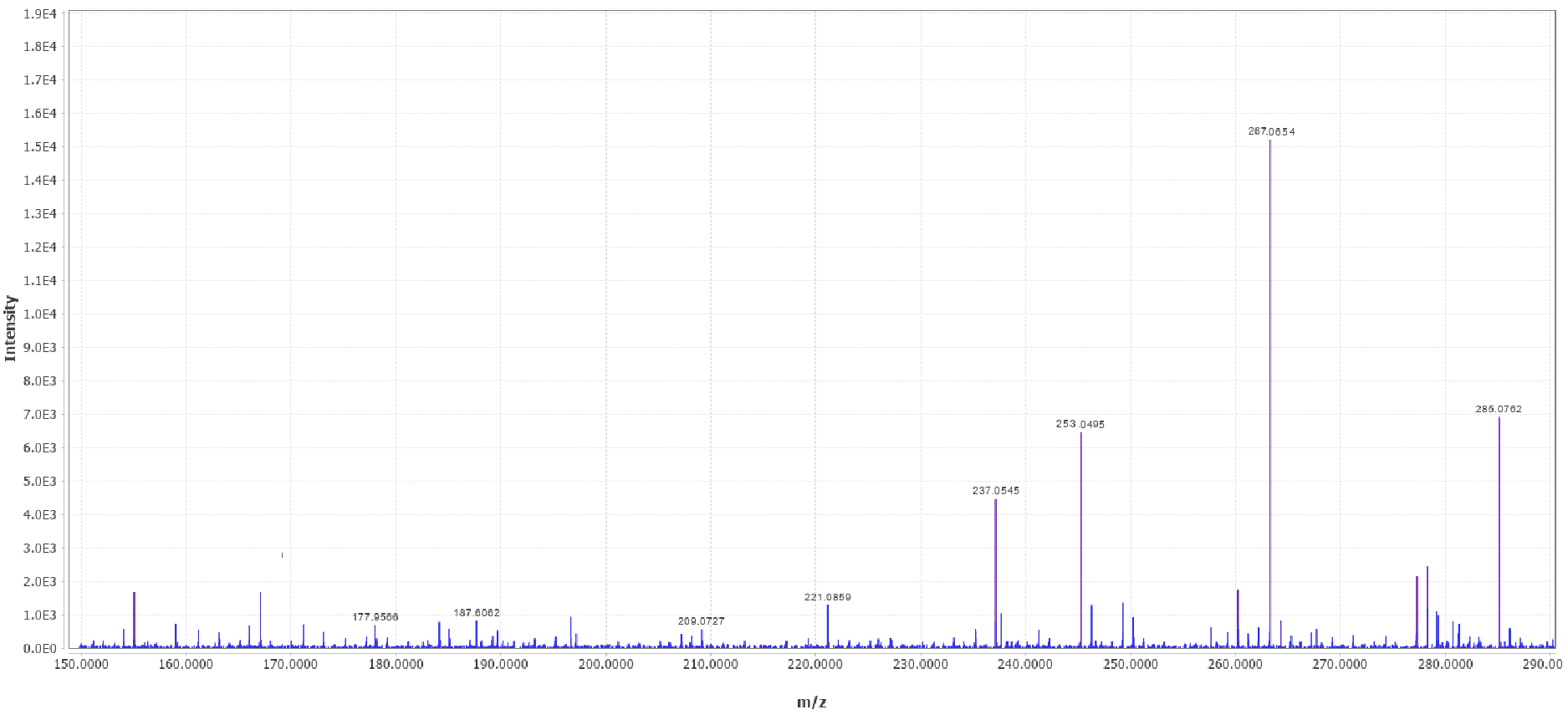


**Figure S12:** MS/MS fragmentation spectrum of physcion (1).


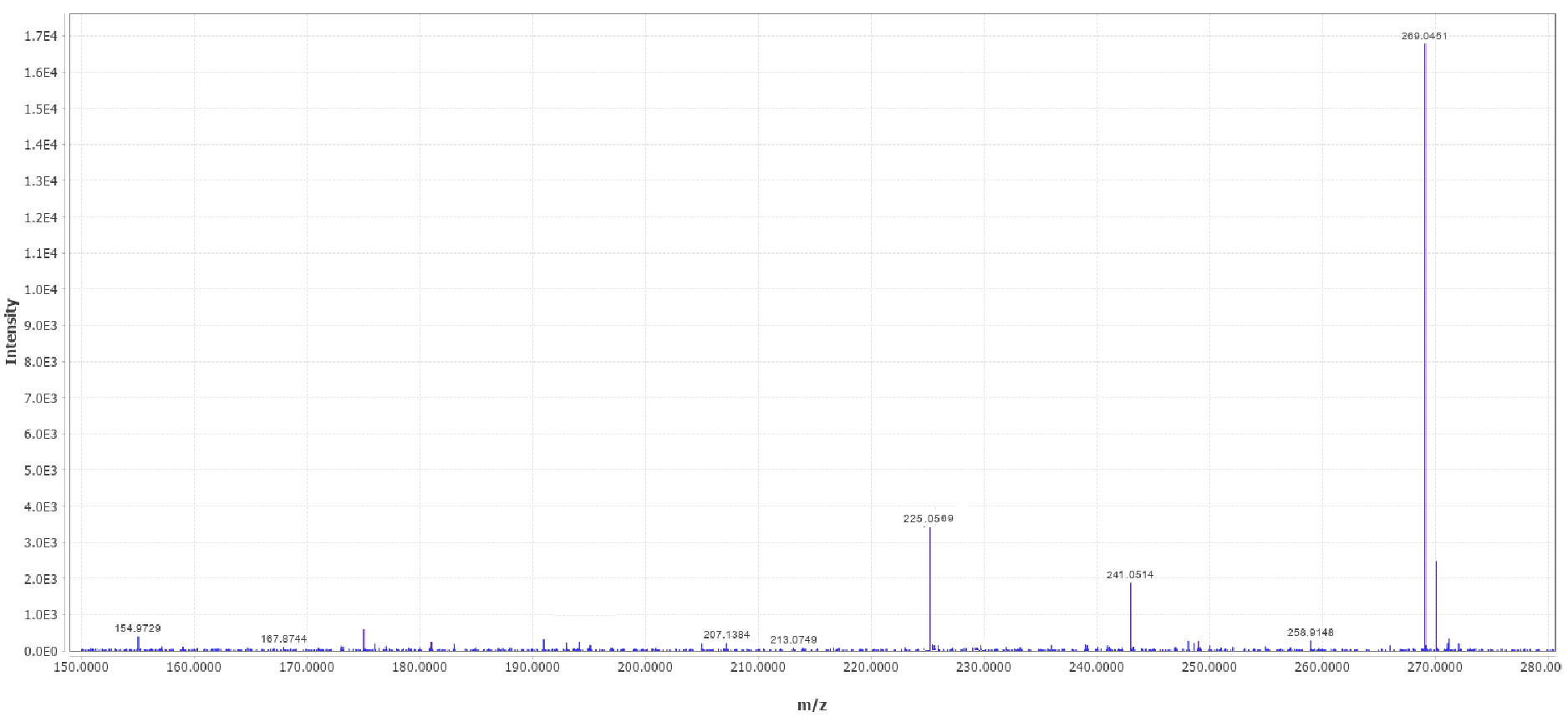


**Figure S13:** MS/MS fragmentation spectrum of emodin (2).


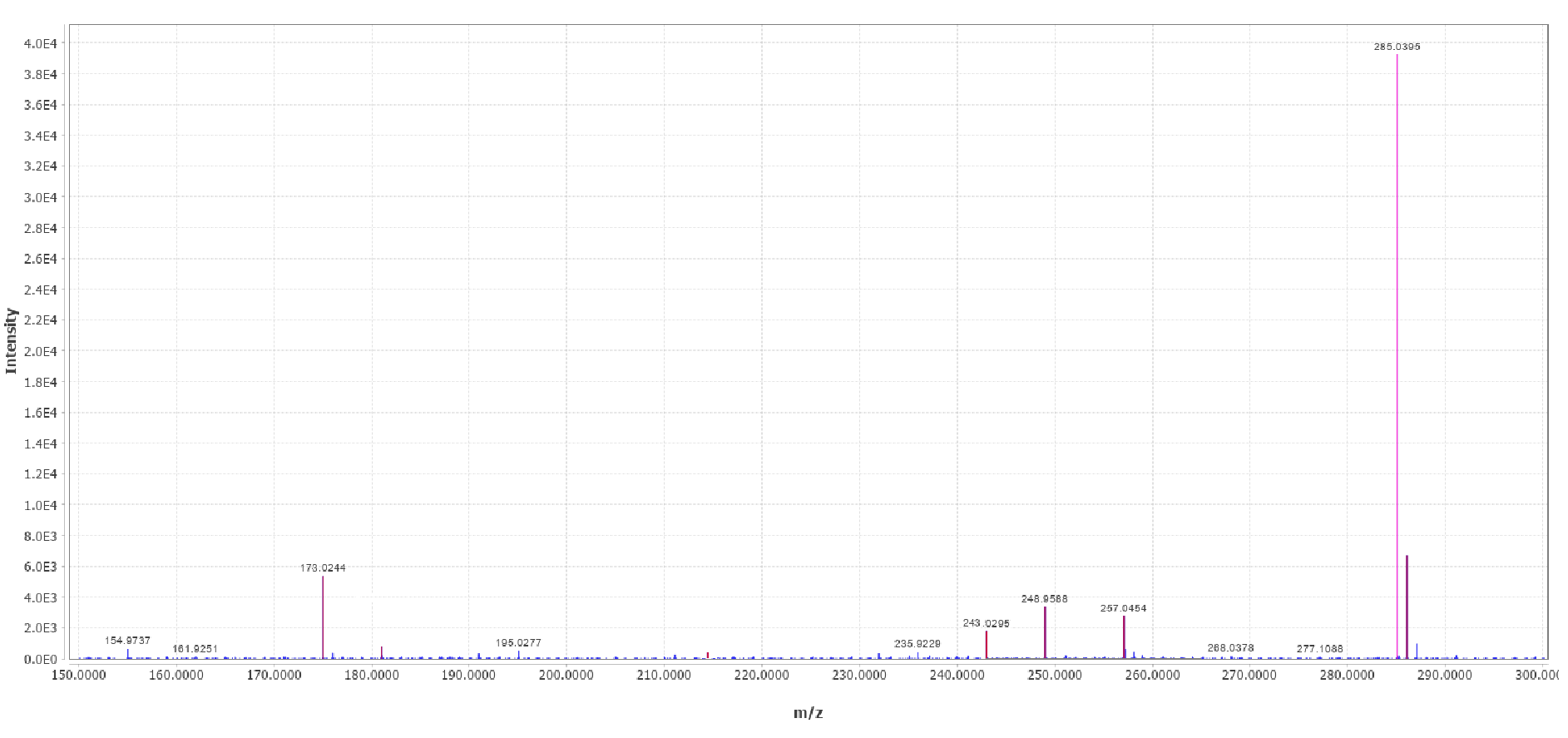


**Figure S14:** MS/MS fragmentation spectrum of katenarin (3).


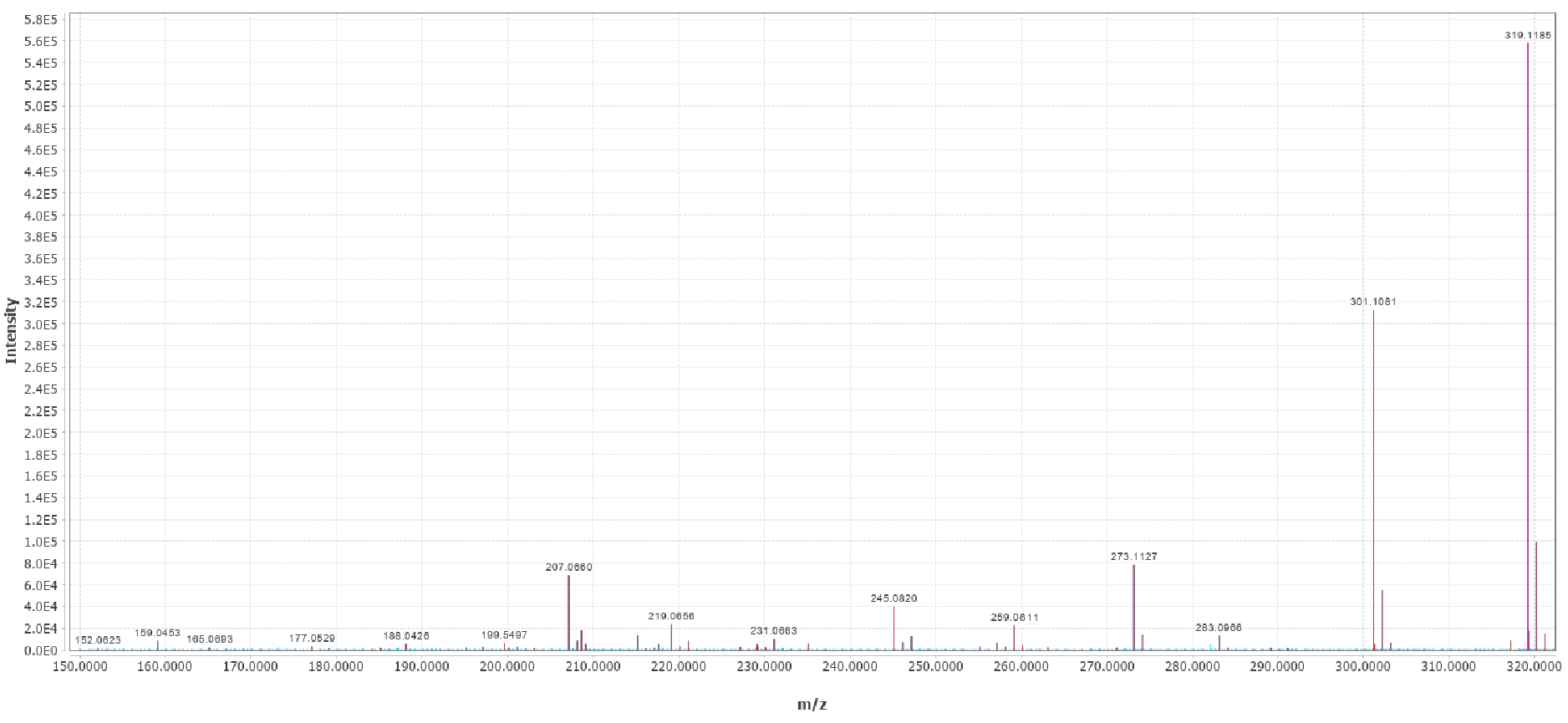
**Figure S15:** MS/MS fragmentation spectrum of Norjavanicin (4).


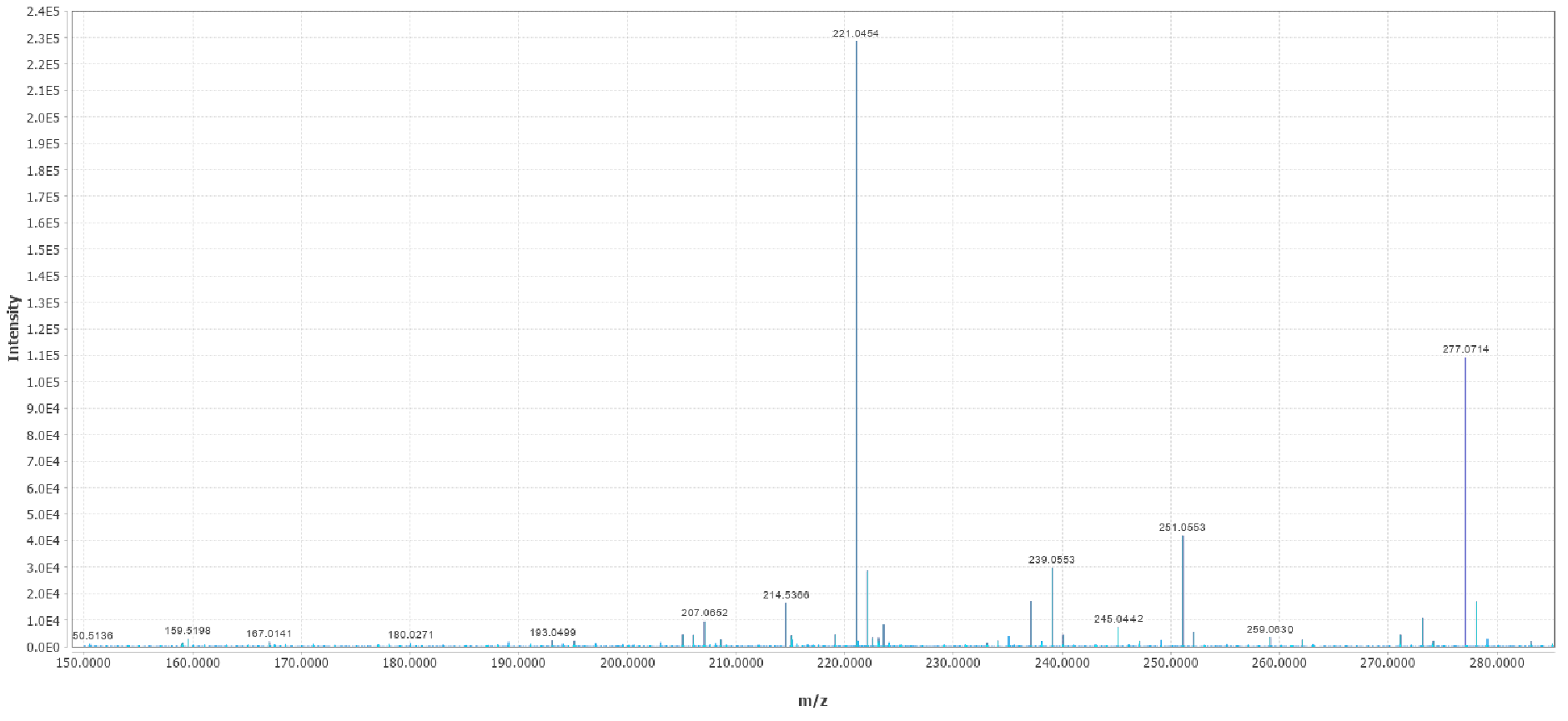


**Figure S16:** MS/MS fragmentation spectrum of Dechlorogriseofulvin (5).


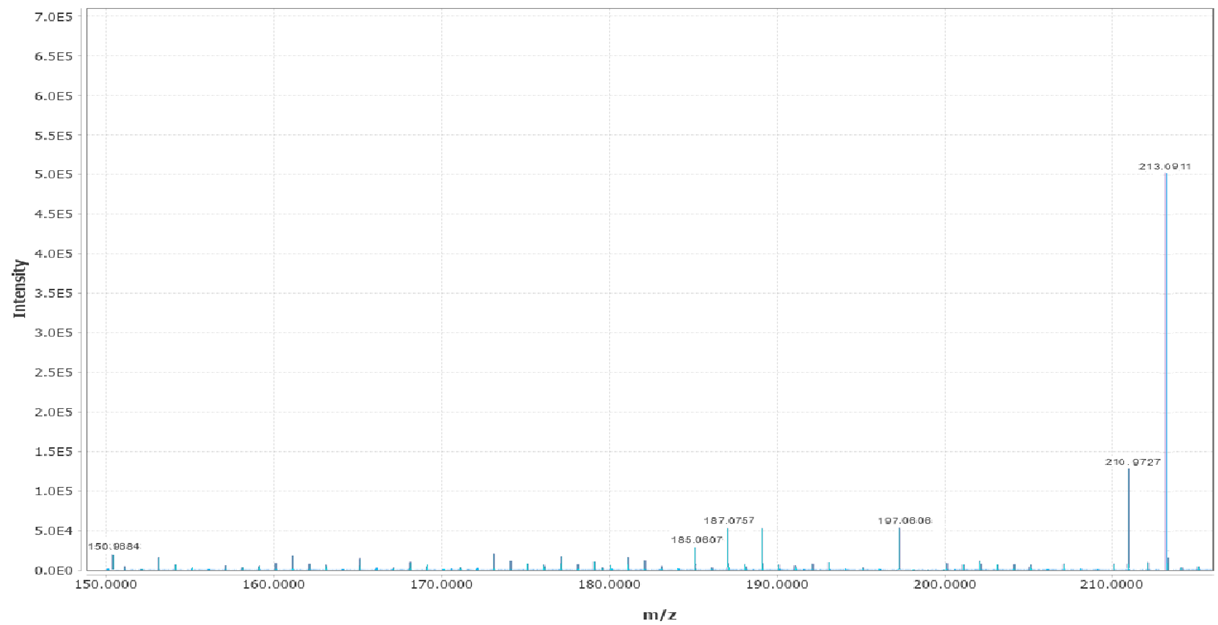


**Figure S17:** MS/MS fragmentation spectrum of Benzyl benzoate (6).


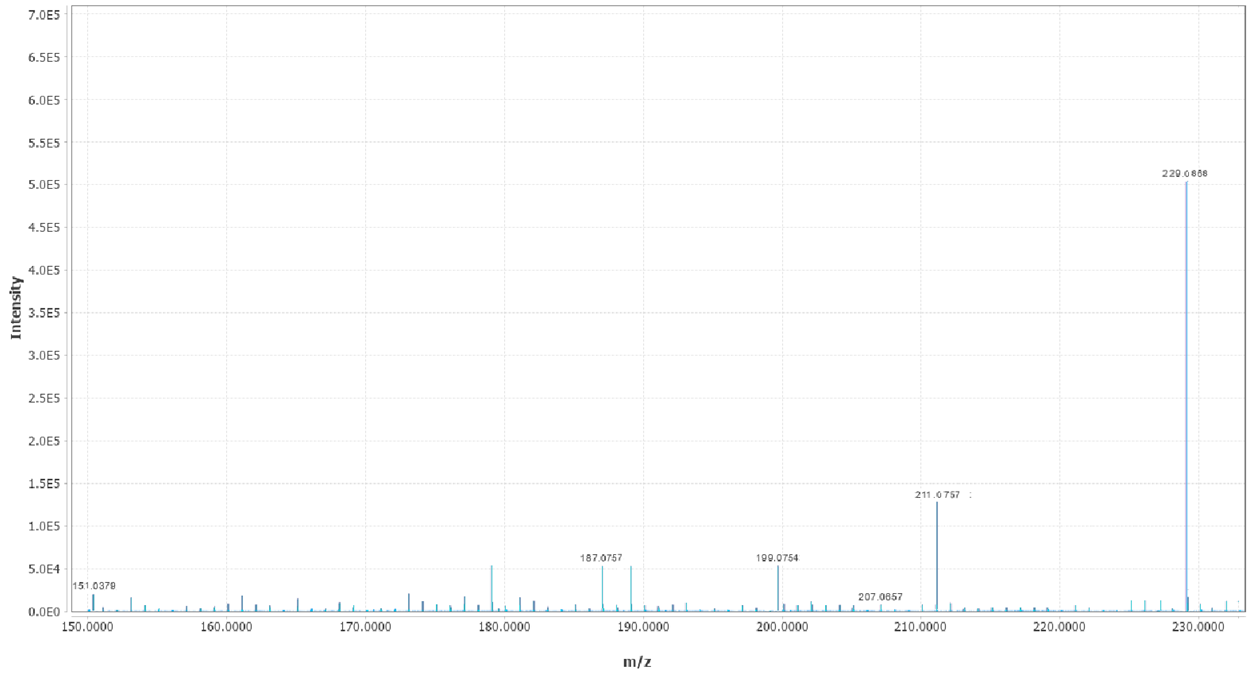


**Figure S18:** MS/MS fragmentation spectrum of 4-hydroxy benzyl benzoate (7).


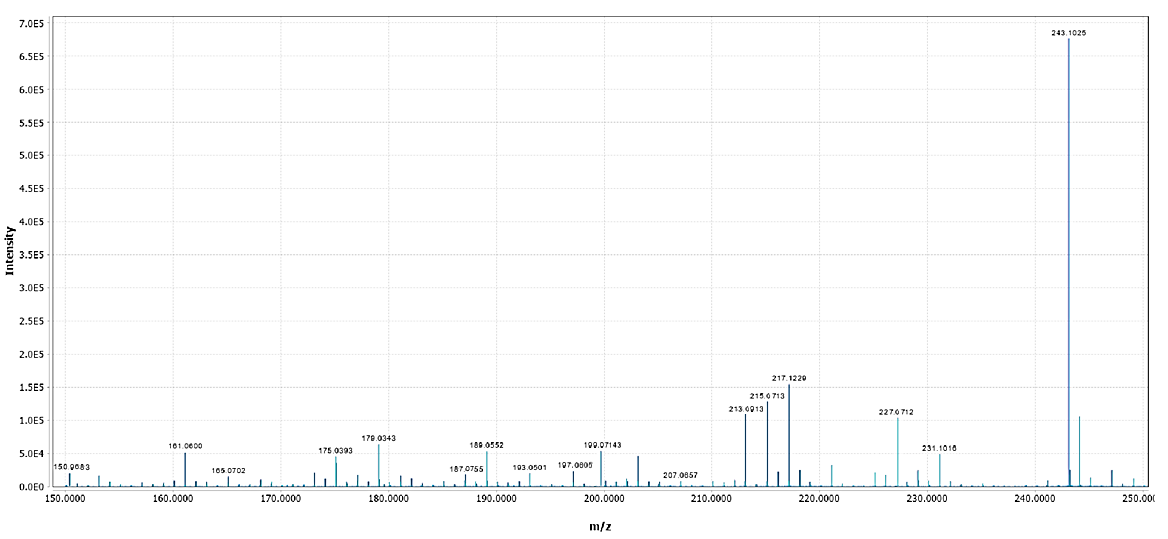


**Figure S19:** MS/MS fragmentation spectrum of Benzyl anisate (8).
